# PARL stabilizes mitochondrial BCL-2 via Nur77-mediated scaffolding as a therapeutic strategy for Parkinson’s disease

**DOI:** 10.1038/s41419-025-08035-8

**Published:** 2025-10-06

**Authors:** Shiyi Yin, Yibo Zhai, Run Song, Jiannan Wu, Yongjiang Zhang, Miao Yu, Hongxia Ma, Mengmeng Shen, Xiaoyi Lai, Weina Jin, Yunqi Xu, Junqiang Yan

**Affiliations:** 1 Key Laboratory of Neuromolecular Biology, The First Affiliated Hospital, College of Clinical Medicine of Henan University of Science and Technology, Luoyang, China; 2 China National Clinical Research Center for Neurological Diseases, Beijing Tiantan Hospital, Capital Medical University, Beijing, China; 3 Department of Neurology, Nanfang Hospital, Southern Medical University, Guangzhou, China; 4 Department of Neurology, The First Affiliated Hospital, College of Clinical Medi-cine of Henan University of Science and Technology, Luoyang, China

**Keywords:** Parkinson's disease, Cell death in the nervous system

## Abstract

Parkinson’s disease (PD) involves both mitochondrial dysfunction and Lewy body pathology. However molecular links between these features remain unclear. Here, we identify Presenilin-associated rhomboid-like protein (PARL) as a Lewy body component, RARL regulates mitochondrial apoptosis via interacting with orphan nuclear receptor Nur77. Clinical profiling revealed reduced plasma PARL levels in 71 PD patients versus controls (*p* < 0.001), which correlated with disease severity. In MPP^+^/MPTP models, PARL depletion amplified BAX activation and caspase-3 cleavage, driving neuronal death. Mechanistically, mitochondrial translocation of Nur77 stabilized PARL-BCL-2 complexes, suppressing apoptosis. AlphaFold2-guided structural modeling uncovered a PARL α-helix essential for Nur77 binding. Disrupting this interface abolished BCL-2 stabilization. *Parl* knockdown exacerbated motor/cognitive deficits in MPTP mice, rescued by Nur77 overexpression. Subcellular tracking demonstrated Nur77 nuclear-cytoplasmic shuttling dynamically regulates PARL-BCL-2 assembly, while co-immunoprecipitation confirmed *Nur77* knockdown dissociates this complex. Our findings define the Nur77-PARL axis as a critical mitochondrial gatekeeper in PD, where PARL serves dual roles as a Lewy body constituent and apoptosis regulator. Reduced circulating PARL levels may reflect disease progression, while the Nur77-PARL structural interface offers a therapeutic target for neuroprotection. This study bridges Lewy body biology with mitochondrial apoptosis. It proposes biomarker-driven strategies to modulate BCL-2-dependent neuronal survival in PD.

**Figure:**
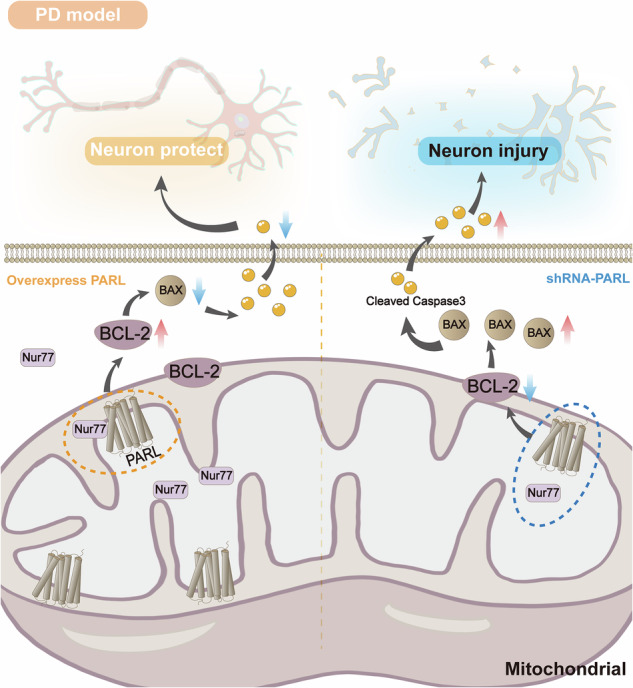
Schematic summary. In normal neuronal cells, PARL can inhibit the release of apoptotic signals by interacting with Nur77. In the MPP^+^-induced PD model, PARL expression is reduced inhibits the apoptosis of dopaminergic neurons, and reduces cell viability. **Mechanistic schema**: **Normal state**: PARL-Nur77 complex stabilizes mitochondrial membrane integrity, inhibiting BCL-2 ubiquitination. **MPP+ injury**: PARL downregulation disrupts Nur77 binding, triggering BAX oligomerization and caspase-3 activation. **Therapeutic rescue**: Nur77 overexpression restores PARL-mediated anti-apoptotic signaling.

Schematic summary. In normal neuronal cells, PARL can inhibit the release of apoptotic signals by interacting with Nur77. In the MPP^+^-induced PD model, PARL expression is reduced inhibits the apoptosis of dopaminergic neurons, and reduces cell viability. **Mechanistic schema**: **Normal state**: PARL-Nur77 complex stabilizes mitochondrial membrane integrity, inhibiting BCL-2 ubiquitination. **MPP+ injury**: PARL downregulation disrupts Nur77 binding, triggering BAX oligomerization and caspase-3 activation. **Therapeutic rescue**: Nur77 overexpression restores PARL-mediated anti-apoptotic signaling.

## Introduction

Parkinson’s disease (PD) is a multifaceted neurodegenerative disorder. It is characterized by the progressive manifestation of motor and non-motor symptoms [1–3], primarily driven by the selective degeneration of dopaminergic neurons in the substantia nigra pars compacta (SNpc) [4–6]. Although genetic and biomarker studies have advanced our understanding of PD risk factors [7], the precise mechanisms underlying dopaminergic neuron loss remain elusive. While mitochondrial dysfunction and apoptosis are recognized as central drivers of neurodegeneration [8–11], the molecular mechanisms regulating mitochondrial integrity in PD remain incompletely understood.

Emerging evidence implicates BCL-2 family proteins as critical arbiters of neuronal fate, balancing pro-survival (e.g., BCL-2) and pro-apoptotic (e.g., BAX) signals through dynamic interactions [12]. In PD, dysregulation of BCL-2 family proteins-critical regulators of mitochondrial outer membrane permeability-triggers cytochrome *c* release and subsequent caspase activation [13]. Notably, BCL-2 itself exhibits context-dependent roles, acting as both an anti-apoptotic factor and a potential pro-death signal when modulated by binding partners [14]. The interactions among family members regulate the mitochondrial outer membrane permeability, leading the release of caspase-activating factors into the cytoplasm and ultimately leading to cell death [15]. HAX1 exhibits anti-apoptotic activity and shares structural similarities with members of the BCL-2 family, including the presence of BH1- and BH2-like domains, as well as a carboxy-terminal transmembrane domain [16].

Presenilins-associated rhomboid-like protein (PARL) contains six or seven transmembrane segments forming a helical bundle within the membrane. It participates in various biogenesis processes [17, 18]. The mitochondrial protease PARL emerges as a key regulator of apoptotic signaling, with its association to Lewy bodies suggesting pathological relevance [19]. Intriguingly, PARL, a mitochondrial intramembrane protease, interacts with HS1-associated protein X-1 (HAX-1) to regulate apoptosis by influencing HtrA2 processing and mitochondrial integrity, maintaining mitochondrial cristae architecture, preventing cytochrome c release, and modulating BCL-2 stability through mechanisms that remain poorly defined [20] [16, 21]. The Oligomerization of B-cell lymphoma-2 (BCL-2) family protein BAX activates the inherent apoptotic pathway [22], and the BCL-2/BAX/Cleaved Caspase-3 signaling pathway controls cell survival and apoptosis and is implicated in the occurrence and development of PD [23–25]. Despite evidence linking PARL to Lewy body pathology, its role in BCL-2-mediated apoptotic signaling in PD remains unexplored.

Recent work reveals that Nur77 binding drives BCL-2 phase separation, which creates membrane-proximal condensates essential for apoptotic control [19]. Nur77, a member of the orphan nuclear receptor transcription factor superfamily, is involved in cell differentiation, apoptosis, metabolism, and mitochondrial homeostasis, playing a critical role in cell homeostasis and pathophysiology [26–28]. Nur77 adds further complexity, shuttling from the nucleus to mitochondria under stress to modulate BCL-2 function [29]. Nur77/BCL-2 is involved in apoptosis of various tumor cells [19, 30]. In tumor cells, Nur77 translocates from the nucleus to the mitochondria and inhibits the anti-apoptotic function of BCL-2, thereby protecting tumor cells from apoptosis [31, 32]. The interaction between Nur77 and BCL-2 can transform BCL-2 from an anti-apoptotic factor to a pro-apoptotic factor. This transformation indicates that Nur77 participates in the apoptotic pathway of tumor cells [33]. Our prior work revealed a neuroprotective role for Nur77 in PD models [34]. This duality highlights the need to investigate Nur77’s regulatory interplay with PARL and BCL-2 in dopaminergic neurons.

Using PD patient samples and experimental models, we demonstrated that PARL deficiency disrupted Nur77-mediated BCL-2 stabilization, leading to mitochondrial permeabilization and caspase-3 activation. Crucially, Nur77-PARL binding enhances BCL-2’s anti-apoptotic conformation through a scaffolding mechanism, which functions differently from PARL’s proteolytic activity. These results position the disruption of the PARL-Nur77-BCL-2 axis as a novel target for neuroprotective strategies in PD.

## Materials and methods

### Study design and participants

This cross-sectional study enrolled 71 idiopathic PD patients (mean age 68 ± 11 years; 37 males/34 females) and 58 age-/sex-matched healthy controls (31 males/27 females) from the First Affiliated Hospital of Henan University of Science and Technology (July 2022–March 2024). PD diagnosis followed UK Parkinson’s Disease Society Brain Bank criteria [35]. This study was approved by the Ethics Committee of the First Affiliated Hospital of Henan University of Science and Technology, and the patient’s informed consent was obtained.

#### Exclusion criteria

(1) PD patients with neurological diseases, such as sequelae of cerebrovascular disease, psychosis, history of definitive encephalitis, or history of brain injury other than PD [35]; (2) PD patients suffering from diabetes, malignancy, kidney, liver, heart failure, severe anemia, or any other acute or chronic debilitating or fatal disease/status; (3) PD patients with eye diseases, including cataracts, glaucoma, amblyopia, etc; (4) PD patients who had infectious diseases during the study or who used antibiotics, steroid hormones or antipsychotic drugs.

### Ethics statement

The study was approved by the Ethics Committee of First Affiliated Hospital of Henan University of Science and Technology. It was conducted according to the principles expressed in the Declaration of Helsinki. The patients gave informed consent for the investigation, with verbal consent specifically obtained for the NMSS, MMSE, and UPDRS Ⅲ measurements. All those measurements were conducted anonymously in this study. Since those measurements benefit the therapies of PD involved in this study, the ethics committee approved that procedure and hopes they will become routine in PD diagnosis and treatment. The consent for the measurement of plasma PARL was written.

### Peripheral blood collection

The 5 mL of venous blood (heparin anticoagulation) of PD patients was collected on an empty stomach in the morning. Samples were centrifuged at 3000 rpm for 10 min in a centrifuge within 1 h, and ELISA was used to determine PARL.

### ELISA

ELISA kits (PARL) were procured from ABEBIO CN. Add 100 µl of standard and sample to the wells separately. Cover with an overlay, then incubate for 2 h at 37 °C away from light. Wash 3 times with washing solution. Next, add 100 µl of diluted Biotin-Conjugate, and incubate at 37 °C for 1 h in the dark. Wash thoroughly and repeat 3 times. Then add 100 µl of diluted Streptavidin-HRP, and incubate for 1 h at 37 °C away from light. Wash 5 times. Add 100 µl of Substrate Solution and incubate at 37 °C for 20 min away from light. Finally add 50 µl of Termination Solution and start measuring absorbance at 450 nm, 540 nm, and 570 nm within 5 min when the first four wells of the highest concentration of the standard turn blue.

### Cell culture and treatment

SH-SY5Y cells (SH-SY5Y cells were kindly provided by stem Cell Bank, Chinese Academy of Sciences) were grown in DMEM/F12(Corning, New York, USA) with 10% fetal bovine serum (Gibco, Gaithersburg, USA), 1% penicillin-streptomycin, 1% Sodium pyruvate (Invitrogen 11360070), 1% NEAA (Invitrogen, 11140050). The cells were cultured in a humid environment containing 5% CO_2_ at 37 °C. SH-SY5Y cells were incubated with MPP^+^ (1 mM) (Sigma-Aldrich, St. Louis, USA) and Csn-B (20 μM) (Med Chem Express, New Jersey, USA) for 24 h.

### Cell transfection

Lentiviral particles expressing PARL-shRNA (NM_001005767; GV112; GeneChem, Shanghai, China) or Control-shRNA (NM_018622; GV112; GeneChem, Shanghai, China) were used in the experiments.

### Plasmid transfection

*PARL*-MYC (NM_018622-MYC; GV702; GeneChem, Shanghai, China), Control-MYC (GV172; GeneChem, Shanghai, China), *PARL*-GST1 NM_018622(del1-52aa; GV712; GeneChem, Shanghai, China), *PARL*-GST2 NM_018622(del53-167aa; GV712; GeneChem, Shanghai, China), *PARL*-GST3 NM_018622(del1-167aa; GV712; GeneChem, Shanghai, China). *Nur77*-Flag (GOSE0330347-Flag; CV702; GeneChem, Shanghai, China), Control-Flag (GOSE0330347-Flag; CV702; GeneChem, Shanghai, China). shRNA designed to specifically silence *PARL*, *Nur77* and scrambled shRNA(control shRNA) were purchased from GeneChem. The sequences of shRNA against *PARL*were as follows:5ʹ-GCGGACAATGATCAGATATTT-3ʹ and 5ʹ-CTGCTATTTGGCAATATGAA-3ʹ. Thesequences of shRNA against Nur77 were as follows: 5ʹ-CCTTCAAGTTCGAGGACTT-3ʹ. The overexpression plasmids and shRNA constructs were transfected into SH-SY5Y cells according to the manufacturer’s instructions.

### Brain stereotactic injection

The *Parl* knockdown mouse model was established by lentivirus shRNA-*Parl*. The mice were divided into 3 groups: (1) sham operation group (sham), (2) scramble-shRNA group (shRNA), and (3) *Parl* knockdown group (sh*Parl*). The sham group was subjected to craniotomy and subsequently administered with a vehicle solution but not injected with drugs. Mice were anesthetized with isoflurane, placed on a stereotaxic injection device, and their skulls were exposed at the bregma. Injection of 3 μL of viral vector, coordinates anteroposterior +1.1, mediolateral +1.5, and dorsal-ventral –3.5 relative to bregma and at a rate of 0.30 μL/ min for 10 min. After the injection, the needle was retained in place for 10 min before suturing. 8 weeks after injection, conduct behavioral experiments, followed by sacrifice for immunofluorescence or immunohistochemistry analysi.

### Animals and MPTP treatment

C57BL/6 J male mice, aged 6–8 weeks, were acquired and maintained under controlled conditions of temperature (approximately 22 °C) and humidity (approximately 50–60%). The lighting schedule was set to a 12-h light:12-h dark cycle. The mice had ad libitum access to standard rodent chow and water. All experiments involving animals were approved by and conformed to the guidelines of the institutional animal care and use committee at the Institute of Biophysics of the Chinese Academy of Sciences (Beijing, China). The study adhere to the pertinent ethical principles and guidelines established by Henan University of Science and Technology, ensuring strict compliance with their prescribed standards of conduct and ethical practices. The mice were randomly divided into 6 groups (*n* = 6 per group): (1) sham operation group, (2) sham operation group+MPTP, (3) sham operation group+Csn-B, (4) sham operation group+MPTP+Csn-B, (5) sh*Parl* group +Csn-B, and (6) sh*Parl* group+MPTP+Csn-B. The first group,i.e., the sham operation group was treated with normal saline (i.p.). MPTP was prepared by dissolving it in 0.9% saline. The mice were injected (i.p.) with MPTP (20 mg/kg) once a day to induce PD in the group (3). The third and fifth group were given injections (i.p.) of Csn-B (10 mg/kg) every day for 14 days. The fourth and sixth group was given injections (i.p.) of Csn-B (10 mg/kg) for 14 days and daily injections of MPTP (20 mg/kg) starting from the seventh day. And This study has been approved by the Ethics Committee of the First Affiliated Hospital of Henan University of Science and Technology.

### Western blotting

Cells and tissue homogenates were lysed with RIPA lysis buffer (Beyotime, P0013B, Beijing, China). Proteins were separated by SDS-PAGE(10–12%) and then transferred to polyvinylidene fluoride(PVDF) membranes.(Millipore Immobilon-PSQ). The membranes were blocked with 10% skimmed milk powder. Before adding the antibodies, the blocking solution was diluted 10-fold. The PVDF membranes was incubated overnight at 4 °C with primary antibodies (1:2000) against PARL (Abcam Cat# ab118554, RRID:AB_10903849), Nur77 (Cell Signaling Technology Cat# 3960, RRID:AB_2153738), BCL-2 (Proteintech Cat# 26593-1-AP, RRID:AB_2818996), BAX (Abcam Cat# ab32503, RRID:AB_725631), Caspase3 (Proteintech Cat# 66470-2-Ig, RRID:AB_2876892), Cleaved-Caspase3 (Cell Signaling Technology Cat# 9664/9664 P, RRID:AB_2070042). Primary antibodies (1:5000) against β-actin (Proteintech Cat# 66009-1-lg, RRID:AB_2782959) and HSP60 (Proteintech Cat# 66041-1-Ig, RRID:AB_11041709). The membranes were washed three times with PBST and then incubated at 37 °C for 1 h with a 1:5000 dilution of the secondary antibody (goat anti-rabbit, 1:5000, CWBio, Cat# CW0103, RRID: AB_2814709; or goat anti-mouse, 1:5000, CWBio, Cat# CW0102, RRID: AB_2814710), After incubation, the membranes were imaged using the ChemiDoc XRS Plus luminescent image analyzer (Bio-Rad, Hercules, CA, USA).

### Co-IP

Briefly, cells were harvested and lysed using cell lysis buffer containing 50 mM Tris–HCl(pH 7.5), 150 mM NaCl, 1% Nonidet P-40, 0.5% sodium deoxycholate, and 1% protease inhibitor cocktails(Sigma-Aldrich). The cell lysates were centrifuged, and the supernatant was then incubated overnight at 4 °C with the indicated antibodies and protein-G beads (Thermo Fisher Scientific). The beads were washed at least five times with cell lysis buffer, and the precipitated proteins were used for further Western blot analysis.

### GST-pull-down

Pierce^TM^GST Protein Interaction Pull-Down Kit was procured from Thermo Fisher Waltham USA. Prepare the washing solution by mixing TBS and Pull-Down lysis solution at a 1:1 ratio. Subsequently, transfer 50 μl of the resin into a rotating column. Added 400 μl of washing solution, mixed thoroughly, placed it into collection tube, and centrifuged at 1250 × *g* for 1 min. Repeat this washing step three times in total. Approximately 500 μl of the GST-tagged fusion protein was placed in a rotating column and incubated overnight at 4 °C. Then, the resin was washed three times. Prey Protein Preparation: 500 μl of prepared prey protein was added to the rotating column, and incubated overnight at 4 °C on the rotating platform, and then 400 μl of washing solution was added and washed five times. Bait-Prey Elution: Prepare an appropriate amount of glutathione elution buffer and adjust its pH to 8.0 with NaOH. Reassemble the column by replacing the bottom and top caps. Add 250 μl of elution buffer and incubate on the rotating platform for 10 min. Then, remove both caps and centrifuge at 1250 × *g* for 1 min The effluent was collected and labeled as eluent, followed by WB analysis.

### TUNEL assays

Apoptosis was detected according to the manufacturer’s instructions. Adherent cells cultured in 12-well plates were washed with PBS. The cells were then fixed with 4% paraformaldehyde for 30 min. After washing with PBS, 0.3% Triton X-100 was added and incubated at room temperature for 5 min. The cells were then incubated at 37 °C in the dark for 60 min with 50 μl TUNEL detection solution, followed by washing three times with PBS or HBSS. Apoptotic positive cells, indicated by fluorescence emission at the specific wavelength, were observed using a fluorescence microscope.

### Transmission electron microscopy

A sufficient number of cell precipitates were collected and fixed in a 1.5 ml centrifuge tube containing 2.5% glutaraldehyde overnight. Afterwards, the samples were rinsed with PBS three times, each for 15 min. The samples were fixed with 1% osmic acid solution for 1–2 h. After being rinsed with PBS 3 times, the samples were placed in 30%, 50%, 70%, and 90% ethanol for dehydration. The samples were dehydrated twice with 100% ethanol and twice with 100% propanol, each time for 15 min. After embedding, the samples were cut into using a Leica UC7 ultramicrotome and then mounted on copper mesh grids. The sections were stained with uranium acetate for 8–15 min, followed by lead citrate staining for 5–10 min. Finally, the samples were dried using a HITACHI HT7800 drying machine.

### Immunohistochemistry

The mice were anesthetized with 2% isoflurane.They were then perfused with normal saline and 4% paraformaldehyde. The brain was removed and dehydrated with a series of 15% and 30% sucrose solutions at 4 °C for 48 h. The brain tissue was then cut into 20 μm thick sections on a cryostat (Microm HM 525, Thermo, CN). Brain sections were washed in 0.01 M phosphate-buffered saline (PBS), blocked with 5% fetal bovine serum albumin (BSA) containing 0.1% Triton X-100 for 1 h, and incubated with TH (25859-1-AP, 1: 500, proteintech) at 4 °C overnight. The next day, the slices were washed three times with PBS and then incubated with an HRP-labeled secondary antibody. All images were observed by an LSM 780 confocal microscope (Zeiss, Oberkochen, Germany). Brain slices with the same anatomical structure were selected from each mouse. Two researchers, blinded to the treatment groups, counted the number of TH-positive cells bilaterally in the substantia nigra pars compacta (SNpc) region across six adjacent sections.

### Immunofluorescence

Cell slides or brain tissue sections were fixed with 4% paraformaldehyde.Then, they were permeabilized with 0.25% Triton X-100 and blocked with goat serum for 30 min. After overnight incubation at 4 °C with primary antibodies against Nur77 and TH (1: 500), cells were washed with three times with PBS., Then, fluorescent secondary antibodies were added and incubated at 37 °C for 1 h. Wash the samples with PBS three times each for 5 min. Then stain with DAPI and observe with an LSM 780 confocal microscope (Zeiss, Oberkön, Germany). Image J software was then used for analysis and statistical evaluation.

### Open field test

Bring the mice to the test site three hours in advance to allow them to adapt to the environment. Before placing each mouse, ensure that the box is clean and odorless. An automated Flex field / open field activity system monitored the mice in a 50 × 50 × 30 cm box. The color of the internal and bottom sides of the behavioral box is white and the box is divided into central area and peripheral region. The mice’s free movement was recorded over a 15-min period. Data processing was then completed on the corresponding computer.

### Elevated plus maze test

The mice were tested for anxiety-like behavior using an elevated plus maze 50 cm from the ground. Each mouse was placed in the central area of the maze, facing the open arms. The entries and exits of mouse into the open arm within 10 min was recorded by an imaging system.

### Rotarod test

Before the formal experiment, the mice were trained for three consecutive days, three times per day. During the formal test, the rotation speed was gradually increased from 0 rpm/min to 6 rpm/min to observe the latency to fall from the rotameter. If a mouse falls, the latency to fall and the rotational speed were recorded. Mice that remained on the rotating rod after 600 were removed, and their time was recorded as 600 s. Each mice were tested three times at 1-h intervals, and the average latency to fall was calculated for statistical analysis.

### Morris water maze test

A black circular flume, 120 cm in diameter and 50 cm height, was used for the water maze. Water was mixed with milk to lighten its color compared to the mice, and the water temperature was maintained at 28 °C. The water depth is about 2 cm higher than that of the platform with a diameter of 10 cm, about 30 cm from a reference point. The device is divided into five regions. The platform was located in area A, while areas B, C, D and E were in a counterclockwise direction. On the first day of the experiment, each mouse was gently placed in the water facing the c-zone wall and observed for 90 s. If the mice were not found within the specified time range, the mice were induced to the platform and observed for 20 s [36].

### Statistical analysis

Statistical analysis was performed using GraphPad Prism 8 software (GraphPad Software, Inc., La Jolla, CA, USA). Data are presented as the mean ± standard error of the mean (SEM) and were collected and analyzed in a blinded manner. Statistical comparisons between groups were conducted using Student’s *t* test or one-way ANOVA with Dunettee’s post hoc test. *P* value of ≤0.05 was considered statistically significant in all experiments, with significance levels denoted as *** for *P* ≤ 0.001, ** for *P* ≤ 0.01, and * for *P* ≤ 0.05. Absence of asterisks indicates non-significance (*p* > 0.05). GraphPad Prism 8.00 (GraphPad Software, San Diego, CA, USA, www.graphpad.com) was used to generate graphs and analyze data.

## Results

### PARL expression is downregulated in the serum of Parkinson’s disease patients and correlates with cognitive and motor functions

We conducted a cross-sectional study to investigate potential differences in serum PARL levels between PD patients and healthy controls.The study involved 129 participants: including 71 PD patients [37 males (52.1%) and 34 females (48.9%)] and 58 age-matched healthy controls [31 males (53.4%) and 27 females (46.6%)]. The mean ages of the PD patients and healthy controls were 68 ± 11 years (range: 43–87 years) and 67 ± 12years (range: 41–83 years), respectively.

PD patients were clinically assessed using the Hoehn-Yahr (H&Y) staging system, a widely used scale to classify the severity of PD based on motor symptoms and functional disability. The H&Y stages range from 1 to 5. Stage 1 indicates unilateral involvement with minimal disability. Stage 2 represents bilateral involvement without balance impairment. Stage 3 characterized by mild to moderate bilateral disease with postural instability. Stage 4 indicats severe disability, but still able to walk or stand unassisted. Stage 5 reflects a wheelchair-bound or bedridden status unless aided. In our cohort, PD patients exhibited H&Y stages ranging from 1 to 5, encompassing the full spectrum of disease severity.

We collected serum samples from all participants and quantified PARL levels. Preliminary analysis showed significant differences in serum PARL levels between PD patients and healthy controls, suggesting that PARL may serve as a potential biomarker for PD. Further stratification of PD patients by H&Y stage demonstrated a progressive decline in serum PARL levels with increasing disease severity, highlighting a potential correlation between PARL expression and the progression of PD. These findings highlight the importance of PARL in the pathophysiology of PD and its potential as a marker of disease severity.

Demographic details of all 129 participants, including PD patients and healthy controls, are summarized in Table 1. Serum PARL levels were significantly lower in PD patients compared to healthy controls (51.90 vs. 60.04, *p* < 0.001), which suggests a potential role for PARL in the pathophysiology of PD. Additionally, cognitive function measured by the Mini-Mental State Examination (MMSE), was significantly impaired in PD patients compared to controls (20.62 vs. 26.82, *p* < 0.001). The MMSE, a widely used screening tool for cognitive impairment, has a maximum score of 30. Scores below 24 are generally indicative of cognitive dysfunction, with lower scores reflecting more severe impairment. In our cohort, the mean MMSE score for PD patients fell within the range of mild to moderate cognitive impairment, consistent with the known non-motor manifestations of PD. No significant differences in age were observed between PD patients and healthy controls (Table 2), confirming that the observed differences in PARL levels and MMSE scores were not attributable to age-related factors.

**Table 1:** The demographic characteristics and clinical data of the patients were included.

Clinical parameters	PD			Healthy People		
	Mean (SD)	Min	Max	Mean (SD)	Min	Max
Male n (%)	37 (52.1)			31 (53.4)		
Female n (%)	34 (48.9)			27 (46.6)		
Age(years)	67.86 (11.37)	43	87	66.87 (12.15)	41	83
MMSE	20.9 (7.03)	4	30	27.25 (2.16)	21	30
H&Y	2.53 (1.11)	1	5			
Disease duration(years)	12.5 (4.8)	1	18			
UPDRS(Ⅲ)	24.9 (10.17)	6	47

**Table 2:** Comparison of subjects PARL, MMSE, and Age between PD patients and healthy people.

Variable	PD	Control	t	p
PARL (pg/ml)	51.90 ± 1.17^a^	60.04 ± 1.29	4.642	<0.001
MMSE	20.62 ± 0.76^a^	26.82 ± 0.32	7.482	<0.001
Age	66.42 ± 1.41	67.86 ± 1.29	0.571	0.568

(Mean ± SEM).

^a^Compared with the Control group, *p* < 0.001.

To further explore the clinical relevance of PARL in PD, we examined correlations between serum PARL levels and several established clinical measures. A significant negative correlation was observed between PARL levels and the Unified Parkinson’s Disease Rating Scale Part III (UPDRS III), assessing motor symptoms (r = –0.289, *p* = 0.017). This indicates that lower PARL levels are associated with more severe motor impairment. Moreover, a significant negative correlation was found between PARL levels and H&Y stages (r = –0.506, *p* = 0.00007), indicating that reduced PARL expression is linked to advanced disease severity. Conversely, a positive correlation was observed between PARL levels and MMSE scores (r = 0.390, *p* = 0.0008), indicating that higher PARL levels are associated with better cognitive function in PD patients (Table 3)

**Table 3:** Spearman’s rank correlation coefficient (rs) and *P* values between PARL and clinical variables.

Variable		Age	MMSE	H&Y	UPDRS(III)
PARL (pg/ml)	r	−0.268	0.390	−0.506	−0.289
	p	0.137	0.0008	0.00007	0.017

**Fig. 1:**
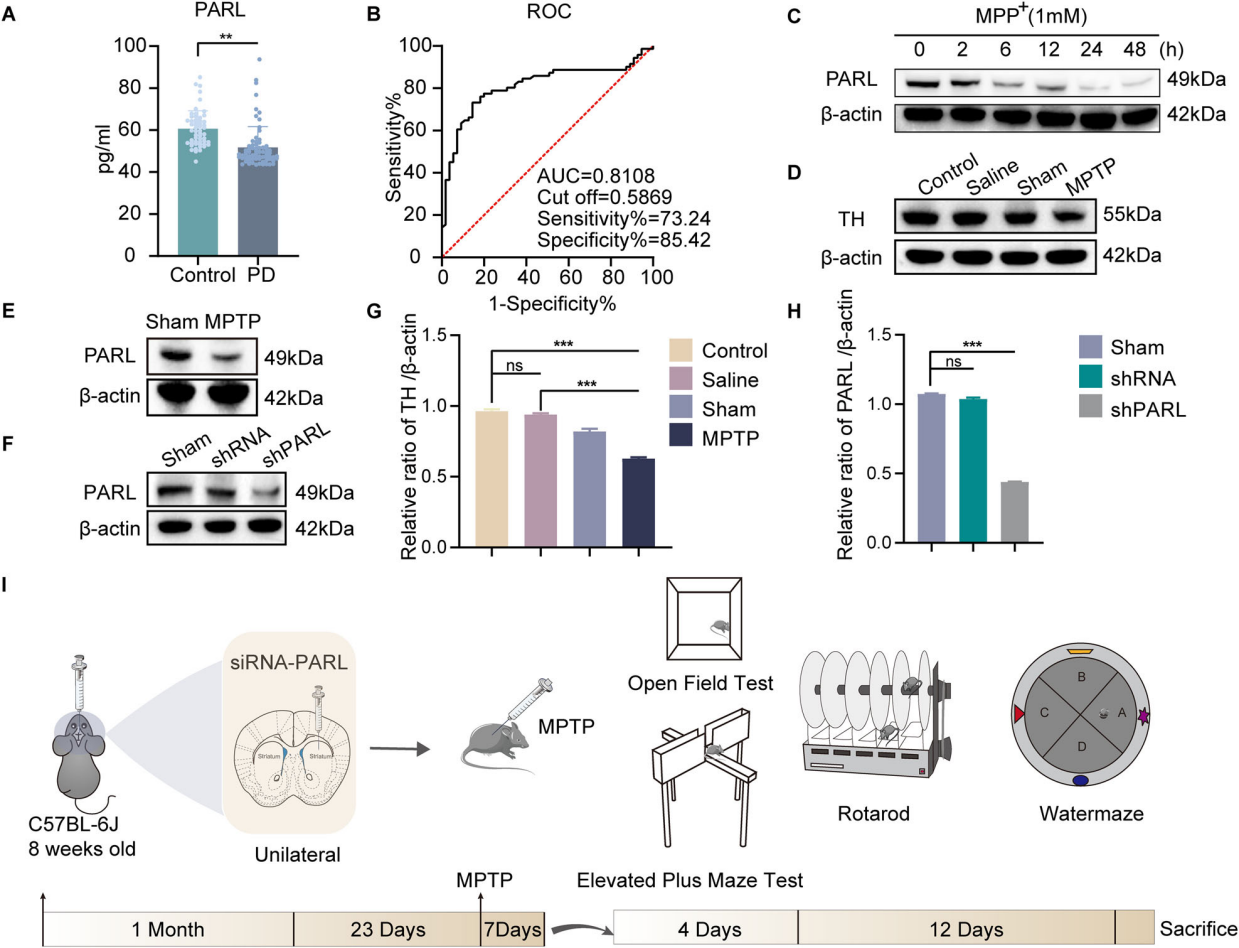
PARL expression decreased in the serum of Parkinson’s disease patients as well as in PD cellular and animal models. ELISA quantification of serum PARL levels in age-matched healthy controls and PD patients. Data presented as mean ± SEM (*p* < 0.001). **A** ROC curve analysis evaluating serum PARL as a PD diagnostic biomarker. **B** SH-SY5Y neuronal viability after MPP^+^ treatment (1 mM) at indicated timepoints (0–48 h). **C** Cell counts normalized to untreated controls. (*n* = 3). **D**, **G** Western blot analysis of tyrosine hydroxylase (TH) in midbrain tissues from MPTP-treated vs. control mice (*n* = 3). **E** PARL protein expression in midbrain lysates (MPTP vs. Control). **F**, **H** PARL knockdown efficiency in C57BL/6 J mice 8 weeks post-lentiviral injection (shRNA vs. control). Data normalized to β-actin (mean ± SD, *n* = 5/group). **I** Timeline of behavioral assessments (rotarod, open field, Morris water maze) performed at 4-week intervals post-MPTP induction.

In this study, we investigated the potential role of PARL (Presenilin-associated rhomboid-like protein) in the pathogenesis of PD. We analyzed serum samples from clinically diagnosed PD patients and age-matched healthy controls. Our findings revealed a significant reduction in PARL levels in PD patients compared to healthy subjects, suggesting a potential association between PARL and PD development. Quantitative analysis demonstrated the marked decrease of PARL protein levels in PD patients. The mean PARL concentration in the PD group was 51.78 ± 9.85 ng/mL, whereas in the control group, it was 60.69 ± 8.53 ng/mL. This reduction was consistent across the majority of PD samples, indicating a robust trend. The boxplot further illustrated the distribution of PARL levels, highlighting the significant difference between the two groups (Fig. 1A). These results suggest that PARL may play a role in the pathological processes underlying PD. We evaluated the diagnostic utility of PARL as a potential biomarker for PD using receiver operating characteristic (ROC) curve analysis, which yielded an area under the curve(AUC) of 0.8108 (95% CI: 0.7322–0.8893), indicating a strong discriminatory power between PD patients and healthy controls (Fig. 1B). At the optimal cutoff value of 0.5869, PARL exhibited a sensitivity of 73.24% and a specificity of 85.42%. These findings suggest that PARL levels in serum could serve as a reliable biomarker for distinguishing PD patients from healthy individuals.

Our findings demonstrate a significant reduction in PARL levels in the serum of PD patients, which correlates with disease severity and exhibits strong diagnostic potential. These results suggest that PARL may critically contribute to PD pathogenesis through mitochondrial dysfunction. Further studies are warranted to explore the mechanistic link between PARL and PD, and its potential as a therapeutic target or biomarker for early diagnosis and disease monitoring.

### *PARL* deficiency exacerbates Parkinsonian behavioral deficits via dopaminergic dysregulation

To investigate the role of PARL in PD, we first examined MPP^+^-treated SH-SY5Y cells and observed a time-dependent decrease in PARL protein expression (Fig. 1C). Consistent with cellular findings, Western blot analysis of midbrain tissues from MPTP-induced subacute PD mice revealed significant downregulation of PARL (Fig. 1E). Concurrently, tyrosine hydroxylase (TH) expression—a hallmark of dopaminergic neuron integrity—was markedly reduced in PD mice (Fig. 1D, G), suggesting a potential link between PARL and dopaminergic dysfunction.

To establish a causal relationship, we generated *Parl*-knockdown mice via stereotaxic injection into the striatum. Successful knockdown was validated by Western blot (Fig. 1, F, H). Behavioral assessments were conducted on both MPTP-induced PD and *PARL*-knockdown mice, with experimental timelines detailed in Fig. 1I.

**Fig. 2:**
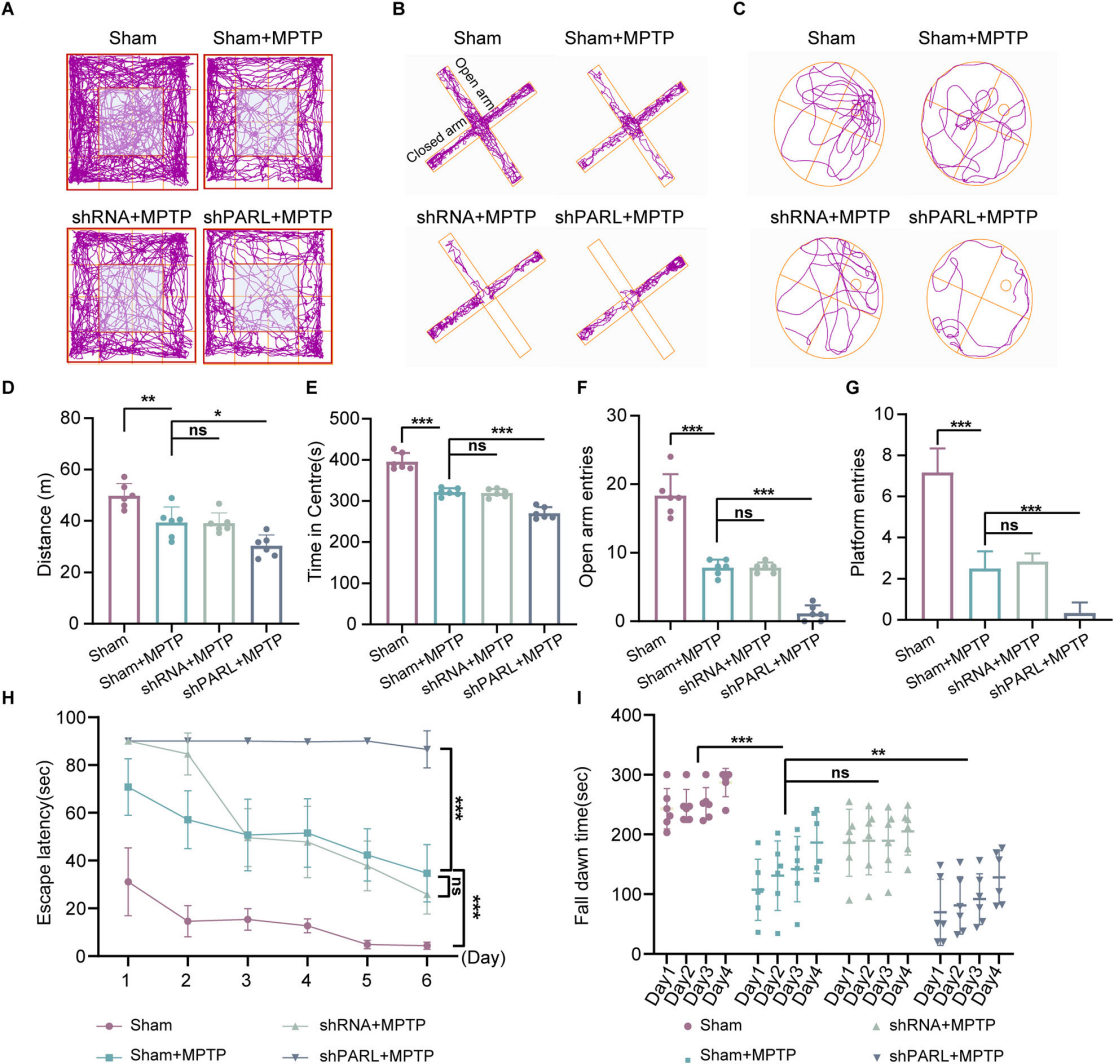
PARL deficiency exacerbates MPTP-induced motor and cognitive deficits in PD mice models. **A**, **D**, **E** Open-field behavior test analysis: Total Track route, Distance and Total Central square time ratio. **B**, **F** Elevated Plus Maze: Test the Total Track route and Mean latency to enter the open arms of the elevated plus maze in a 5-min test period. **C**, **G** Morris water maze test: representative swimming tracks of mice (time to find the platform) in Morris water maze test. The number of entries in the platform was significantly increased in shPARL mice compared to Sham mice on the last day. **H** At the same time, the escape latency was significantly decreased in shPARL mice compared to Sham mice. **I** Latency to fall of various groups assessed by the rotarod test. (ns not significant, ∗*p* < 0.05, ∗∗*p* < 0.01, ∗∗∗*p* < 0.001, one-way ANOVA followed by Turkey’s test for multiple comparisons, *n* = 6 per group. Bars represent mean ± SD).

To evaluate the impact of *Parl*-knockdown on motor and cognitive functions in PD, we conducted a series of behavioral tests: the open field test, elevated plus maze, Morris water maze, and rotarod test. In the open field test, *Parl*-knockdown mice exhibited reduced exploratory activity, spending significantly less time in the central zone compared to controls and MPTP-treated mice (Fig. 2A, D, E). This peripheral preference indicates heightened anxiety-like behavior. In the elevated plus maze, *Parl*-knockdown mice amplified anxiety phenotypes, with knockdown mice spending less time in open arms (Fig. 2B, F). During the Morris water maze test over 7 days, *Parl*-knockdown mice showed impaired spatial learning, evidenced by prolonged latency and fewer platform crossings (Fig. 2C, G, H). Finally, the rotarod test revealed exacerbated motor coordination deficits in *Parl*-knockdown mice, as shown by shorter fall latency (Fig. 2I).

These behavioral alterations were accompanied by a marked downregulation of TH expression in the midbrain, indicating dopaminergic neuron loss. Collectively, these findings underscore the critical role of PARL in maintaining dopaminergic function and suggest that *Parl*-knockdown contributes to the progression of PD-related behavioral and pathological impairments.

### PARL modulates mitochondrial apoptosis via BCL-2/BAX/caspase-3 signaling in MPP^+^-induced Parkinsonian models

To investigate the molecular mechanisms by which PARL regulates apoptosis in PD, we analyzed transcriptomic and proteomic changes in MPP^+^-lesioned SH-SY5Y cells and PD mouse models. High-throughput mRNA sequencing revealed that *PARL* knockdown induced significant downregulation of BCL-2 (log2FC > 0.5, *P* < 0.05) and upregulation of pro-apoptotic genes including BAX and Caspase-3 (Supplementary Fig. 1A). A heatmap visualization further illustrated the differential expression of apoptosis-related genes, including BCL-2, across the experimental conditions (Supplementary Fig. 1B). Comparative analysis of shared and unique genes among the four treatment groups was also performed, highlighting distinct expression patterns (Supplementary Fig. 1C, D). Focusing on the global gene expression profile, we identified a significant reduction in BCL-2 transcript levels in the *PARL*-knockdown group (log2FC > 0.5, adjusted P-value < 0.05) (Supplementary Fig. 1E). Functional enrichment analysis further highlighted apoptosis and neuronal death as key pathways affected by *PARL* downregulation (Supplementary Fig. 1F), aligning with studies showing BCL-2/BAX imbalance as a hallmark of PD-related neurodegeneration.

**Fig. 3:**
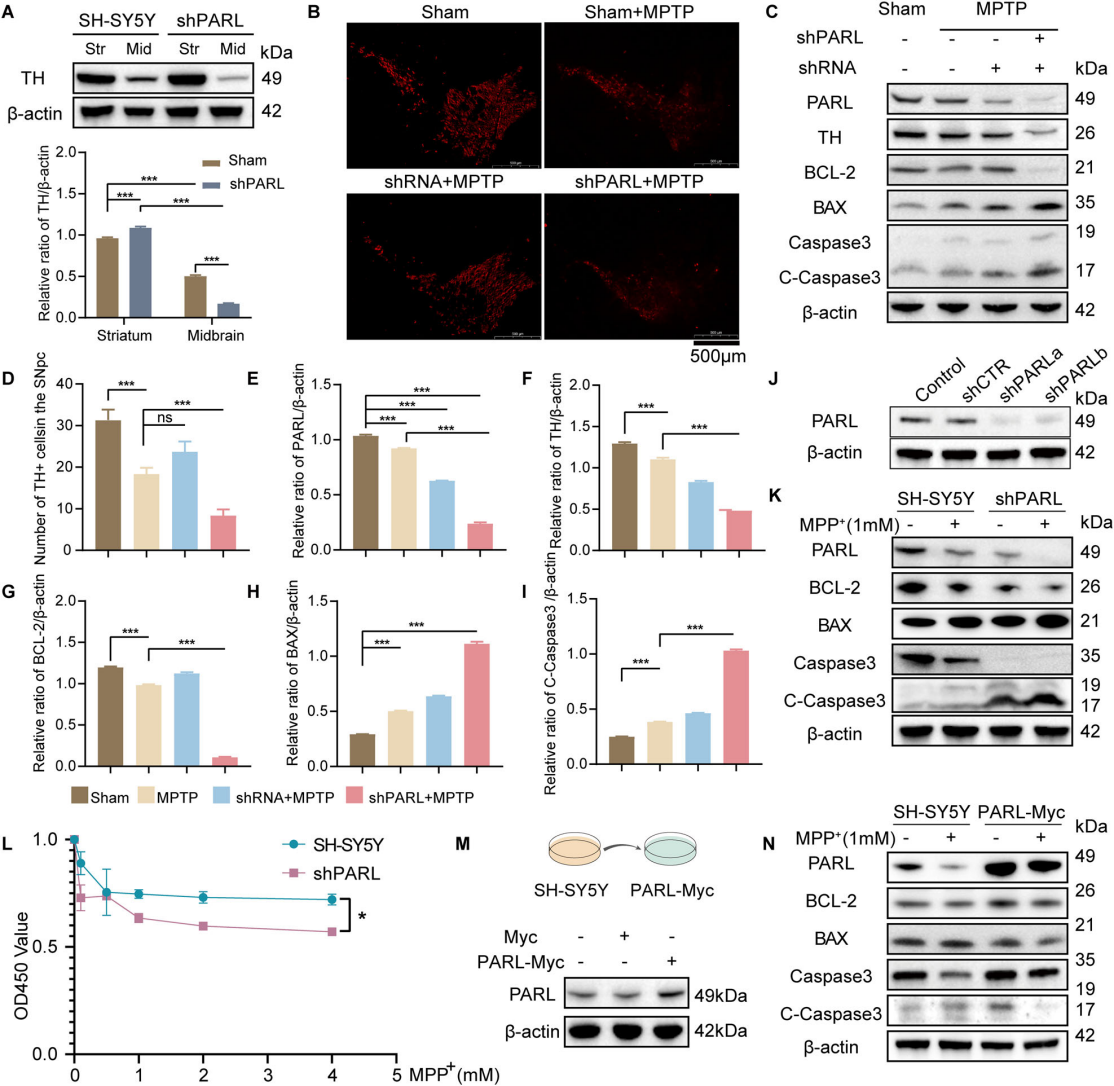
Effect of PARL knockdown and overexpress on apoptosis in PD cellular and animal models. **A** TH expression changes in C57BL/6 J mouse striatum and midbrain 8 weeks post-PARL-shRNA lentivirus injection. Quantitative analysis shows significant TH reduction (***p < 0.001 vs. control, *n* = 3). **B**, **D**TH immunofluorescence (red) in substantia nigra pars compacta (SNpc). PARL knockdown reduced TH+ neurons (**p < 0.01). Scale bar: 500 µm. **C** PARL silencing in MPTP-induced PD mice increased apoptosis markers: BAX/BCL-2 ratio and Cleaved Caspase-3 (C-Casp3) increased (***p < 0.001). β-actin served as loading control. **E**–**I** Quantitative analysis of TH, BCL-2, BAX, Caspase3 and Cleaved-Caspase3(C-Caspase3) protein expressions in different Groups. **J** Successful PARL knockdown in SH-SY5Y cells via shRNA (*p* < 0.001). **K** PARL-deficient SH-SY5Y cells exhibited pro-apoptotic shifts: BAX/BCL-2 ratio and C-Casp3 increased (***p < 0.001). **L** CCK8 assay: PARL knockdown reduced SH-SY5Y viability (***p < 0.001). **M** PARL-Myc overexpression in SH-SY5Y cells. Western blot confirms PARL expression (Myc-tagged) 24 h post-transfection (Lipofectamine 3000, 3 µg plasmid/10⁶ cells). β-actin served as loading control. **N** Rescue of MPP^+^-induced apoptosis by PARL overexpression. Cells co-transfected with PARL plasmid (2 µg/well) for 24 h were treated with MPP^+^ (1 mM, 24 h). Immunoblot analysis shows BCL-2 restoration (vs. MPP^+^ alone), BAX suppression, and Cleaved Caspase-3 (C-Casp3) reduction (**p < 0.01, *n* = 3, one-way ANOVA with Tukey’s post hoc). Representative blot from three biological replicates.

Western blot analysis confirmed that *Parl*-knockdown in PD mice and SH-SY5Y cells reduced BCL-2 expression while elevating BAX and cleaved Caspase-3 (Fig. 3C, E–I). To investigate the role of PARL in the PD cell model, SH-SY5Y cells were transiently transfected to knock down *PARL*, resulting in a marked downregulation of PARL expression post-transfection (Fig. 3, J). We observed a significant downregulation of BCL-2 expression and upregulation of BAX and cleaved-Caspase3 in *PARL*-knockdown cells (Fig. 3, K and Supplementary Fig. 2A–E). These changes in apoptotic markers corresponded with reduced cell viability measured by CCK-8 assays, with *PARL*-knockdown cells exhibiting significantly lower viability compared to control cells (Fig. 3L). To further elucidate the potential protective role of PARL in PD, we employed a *PARL*-Myc construct to overexpress PARL in cellular models of PD. Western blot analysis confirmed successful transfection and overexpression of *PARL*-Myc (Fig. 3M). Overexpression of PARL significant increase the anti-apoptotic protein BCL-2 and decrease the pro-apoptotic proteins BAX and Cleaved Caspase-3 (Fig. 3N). Quantitative analysis further validated these findings, demonstrating statistically significant changes in protein expression levels (Supplementary Fig. 2F–J).

**Fig. 4:**
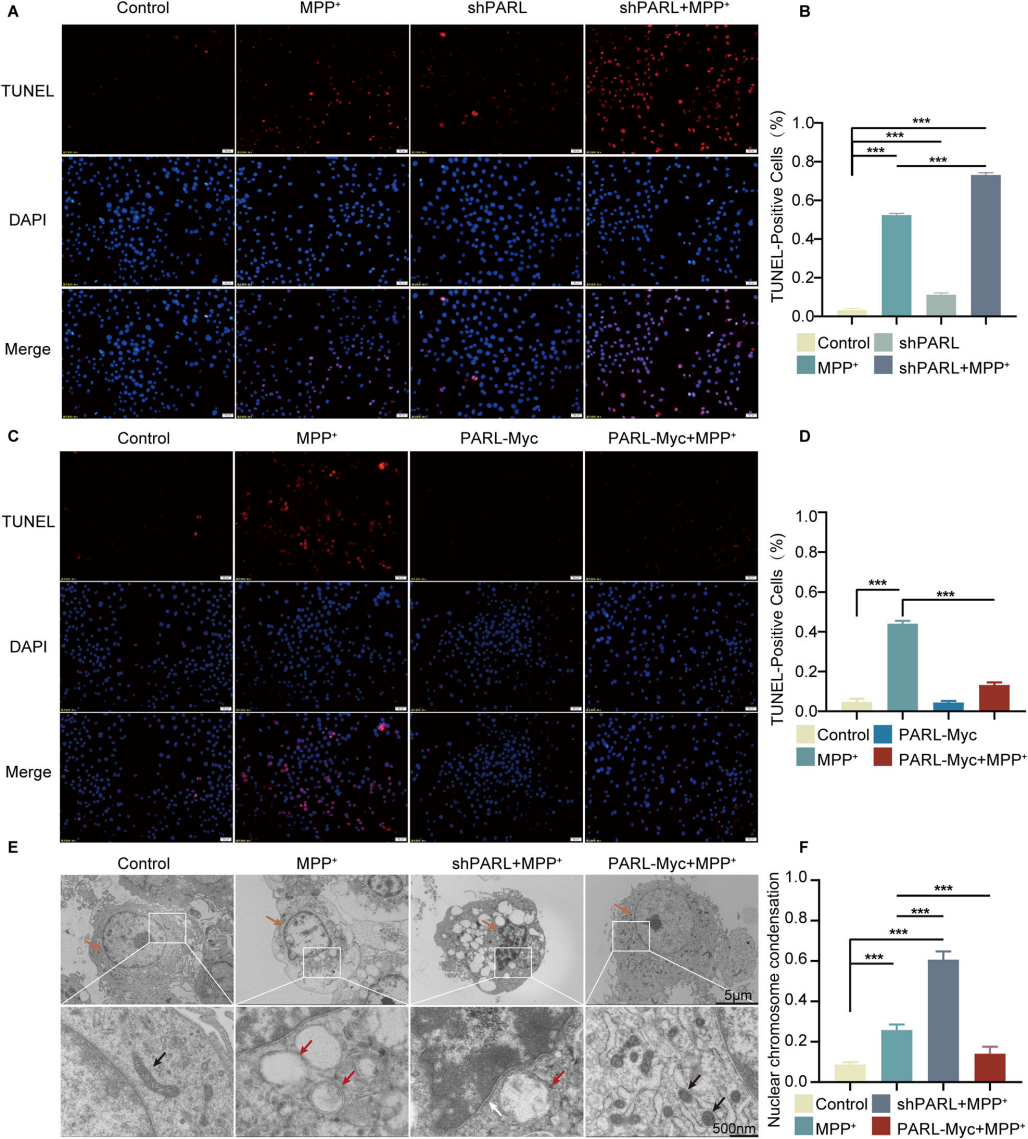
Effect of PARL knockdown and overexpress on apoptosis in PD cellular. **A**, **B** PARL knockdown (via shRNA) significantly increased TUNEL+ cells (red fluorescence), demonstrating enhanced DNA fragmentation and apoptosis in SH-SY5Y cells. Quantitative analysis **B** confirms a significantly **increase** in apoptotic cells compared to controls (*p* < 0.001). **C**, **D** Overexpression of PARL-Myc reduced TUNEL+ cell counts versus knockdown groups (*p* < 0.01), supporting PARL’s role in suppressing caspase-dependent apoptotic pathways. (Bar = 20 μM). **E**, **F** PARL knockdown exacerbated mitochondrial vacuolar swelling (red arrows) and nuclear chromatin aggregation (orange arrows). PARL overexpression partially restored cristae structure (black arrows) and reduced chromatin condensation. Quantification **F** shows 54% reduction in damaged mitochondria (*p* < 0.05). Analyzed by two-tailed Student’s *t* test or one-way ANOVA with Tukey’s post hoc test (ns, not significant, ∗*p* < 0.05, ∗∗*p* < 0.01, ∗∗∗*p* < 0.001 compared with the control group; *n* = 3, mean ± SEM Bar = 20 μM).

To assess the impact of PARL on apoptosis in PD models, we performed immunofluorescence analysis using TUNEL staining. *PARL*-knockdown PD cells showed a significant increase in TUNEL-positive cells compared to controls, indicating enhanced apoptosis (Fig. 4A, B). In contrast, of PARL overexpression in the *PARL*-Myc PD cell model markedly reduced TUNEL-positive cells, suggesting that attenuates apoptotic cell death (Fig. 4C, D). Furthermore, transmission electron microscopy (TEM) was utilized to evaluate mitochondrial morphology. In the PD model group, mitochondria showed significant structural abnormalities, including disappearance of cristae and swelling. *PARL* knockdown exacerbated these mitochondrial defects and was associated with additional cellular changes, such as nuclear shrinkage and nucleoplasmic aggregation. Notably, overexpression of PARL mitigated these pathological alterations, restoring mitochondrial morphology and reducing nuclear abnormalities (Fig. 4E, F). These findings suggest PARL mitigates apoptosis by stabilizing mitochondrial morphology and regulating BCL-2/BAX dynamics. The mechanism consistent with previous studies on cGAS signaling in DNA damage-induced apoptosis.

### Mitochondrial translocation of Nur77 modulate BCL-2/BAX balance via PARL to attenuate apoptosis in Parkinsonian models

**Fig. 5:**
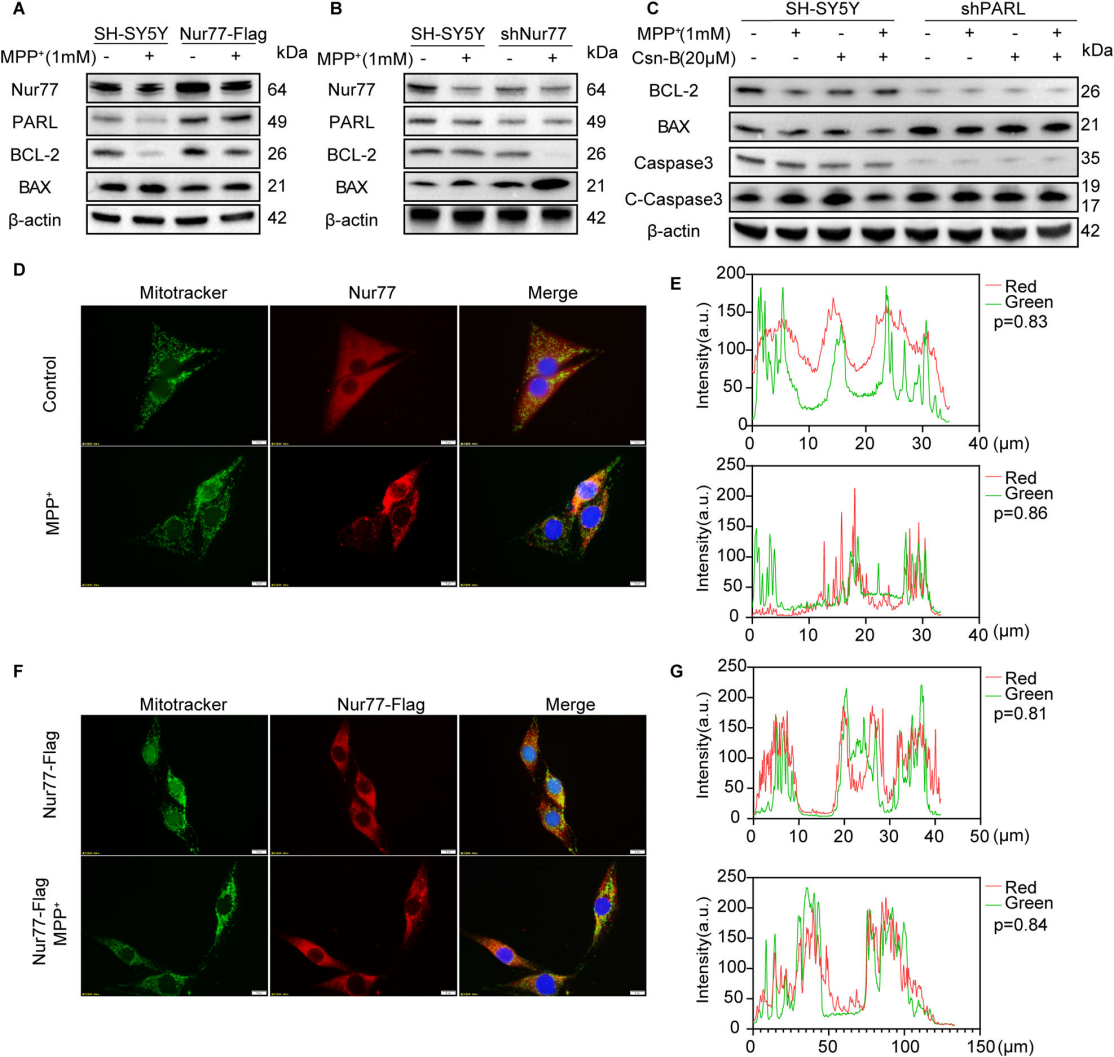
PARL was regulated by Nur77 to alleviate apoptosis in MPP^+^-lesioned SH-SY5Y cells. **A** Western blot analysis of extracts Nur77, PARL, BCL-2, and BAX. Overexpression of Nur77 increased PARL and reduced apoptosis of SH-SY5Y cells. Data are representative of three independent experiments, which gave similar results. **B** SH-SY5Y cells were co-transfected with Nur77 plasmid for 24 h and treated with (or without) MPP^+^ for an additional 24 h. Cell lysates were extracted for immunoblot analysis of Nur77, PARL, BCL-2, and BAX. Representative WB is shown. **C** Nur77 was overexpressed in SH-SY5Y cells with low PARL expression, and the expressions of BCL-2, BAX, Caspase3 and Cleaved-Caspase3 were detected by western blotting. **D**, **E** Fluorescence MitoTracker Green staining in Control and MPP^+^-treated SH-SY5Y cells; mitochondria (GREEN) and Nur77 (RED). Bar = 5 μm. **F**, **G** Fluorescence MitoTracker Green staining in Nur77-Flag and MPP^+^-treated Nur77-Flag cells; mitochondria (GREEN) and Nur77-Flag (RED). Bar = 5 μm. (ns not significant, ∗*p* < 0.05, ∗∗*p* < 0.01, ∗∗∗*p* < 0.001 compared with the control group; *n* = 3, mean ± SEM).

Our previous studies confirmed that Nur77’s translocation from cytoplasm into mitochondria can alleviate mitochondrial damage [37]. To further clarify whether the interaction between PARL and Nur77,we conducted additional experiments and identify Nur77 and PARL interactions using the Protein Interaction Network (Supplementary Fig. 3A). Overexpression of Nur77 in SH-SY5Y cells significantly upregulated PARL and anti-apoptotic protein BCL-2. At the same time, it suppressed the pro-apoptotic BAX (Fig. 5A, Supplementary Fig. 2K–N and Supplementary Fig. 3B). Furthermore, *Nur77* knockdown via shRNA reduced PARL and BCL-2 expression while elevating BAX levels(Fig. 5B and Supplementary Fig. 2O–R), demonstrating that Nur77 positively regulates BCL-2 through PARL-dependent mechanisms.

We employed Csn-B, a Nur77-specific activator, to investigate this pathway [38]. In MPP^+^-treated PD models, Csn-B reversed the induced upregulation of BAX and downregulation of BCL-2 (Fig. 5C and Supplementary Fig. 3E–H). However, this protective effect of Csn-B was abolished in *PARL*-deficient cells, underscoring PARL’s indispensability in Nur77-mediated apoptosis regulation. These findings align with studies showing that Nur77 inhibits mitochondrial apoptosis via BCL-2 modulation, while PARL’s role in mitochondrial integrity further supports its cooperation with Nur77.

Additionally, we examined the subcellular localization of Nur77 after MPP^+^ treatment. Immunofluorescence and subcellular fractionation analyses demonstrated that Nur77 translocated from the cytoplasm to the mitochondrion following MPP^+^ treatment (Fig. 5D, E). This translocation was also observed for Nur77-Flag (Fig. 5F, G), with significantly higher levels of Nur77 detected in the mitochondrial fraction than in the cytoplasm (Supplementary Fig. 3C, D).

Our work identifies PARL as a pivotal mediator of Nur77’s mitochondrial anti-apoptotic activity in mitochondria, acting by regulating BCL-2 and BAX. These findings reveal promising therapeutic opportunities for PD by targeting the Nur77-PARL-BCL-2 axis.

### Nur77 attenuates dopaminergic neuron loss in PD via PARL-dependent regulation of mitochondrial BCL-2/BAX apoptotic signaling

**Fig. 6:**
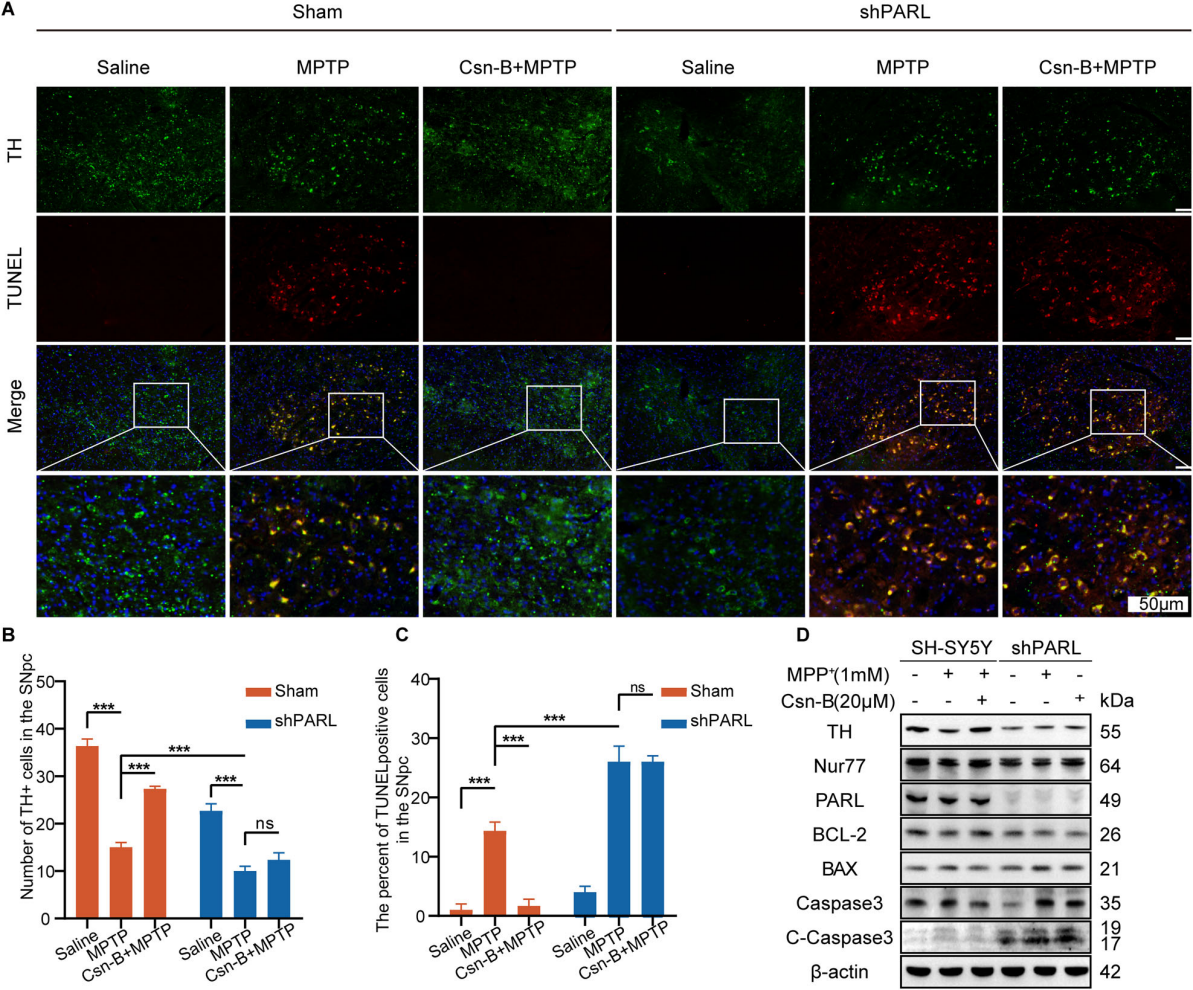
Nur77 overexpression mitigates neurodegeneration in a PD mouse model via PARL-dependent mechanisms. **A** Representative immunofluorescence images of TH (green; tyrosine hydroxylase) and TUNEL (red; apoptotic nuclei) co-staining in the substantia nigra pars compacta (SNpc) of PD mice. Nuclei were counterstained with DAPI (blue). Scale bar: 50 µm. *Top panel*: Individual channels; *bottom panels*: Merged channels. Quantification of TH-positive neurons (**B**) and TUNEL-positive apoptotic nuclei (**C**) in the SNpc across experimental groups. **D** Western blot analysis of TH, PARL, Nur77, BCL-2, BAX, Caspase 3, and Cleaved-Caspase 3 protein levels in midbrain lysates. β-actin was used as a loading control. Low PARL expression abrogates the neuroprotective effects of Nur77 overexpression in PD mice. (ns not significant, ∗*p* < 0.05, ∗∗*p* < 0.01, ∗∗∗*p* < 0.001 compared with the control group; *n* = 3, mean ± SEM).

In *Parl*-knockdown PD mice (generated via AAV-9), we observed a 40% increase in TUNEL-positive apoptotic neurons (*P* < 0.001) and a 55% reduction in TH+ dopaminergic neurons compared to MPTP-treated controls (Fig. 6A–C and Supplementary Fig. 4A). These results indicate enhanced neuronal apoptosis and DA neuron loss in the absence of PARL. To assess the role of Nur77 in this context, we administered Csn-B via intraperitoneal injection. In MPTP-lesioned mice, Csn-B treatment increased midbrain Nur77 levels, reduced TH+ neuron loss, and elevated TH expression, demonstrating its neuroprotective effects. However, these protective effects were abolished in PD mice with altered PARL expression, suggesting that PARL is essential for Nur77-mediated neuroprotection (Fig. 6D and Supplementary Fig. 4B, C). Western blot analysis further revealed that PARL inhibition led to decreased BCL-2 expression and increased levels of the pro-apoptotic proteins BAX and cleaved Caspase-3 in PD mice. Notably, the ability of Csn-B to increase BCL-2 and decrease BAX and Cleaved Caspase-3 expression was significantly impaired in *Parl*-knockdown PD mice (Fig. 6D and Supplementary Fig. 4D–F). These findings suggest that *Parl* knockdown disrupted Nur77’s ability to suppress apoptosis, as shown by reduced BCL-2 and elevated BAX and cleaved Caspase-3 in PD mice.

### Structural and functional elucidation of Nur77-PARL-BCL-2 anti-apoptotic axis in Parkinson’s disease via alphaFold2-guided modeling

**Fig. 7:**
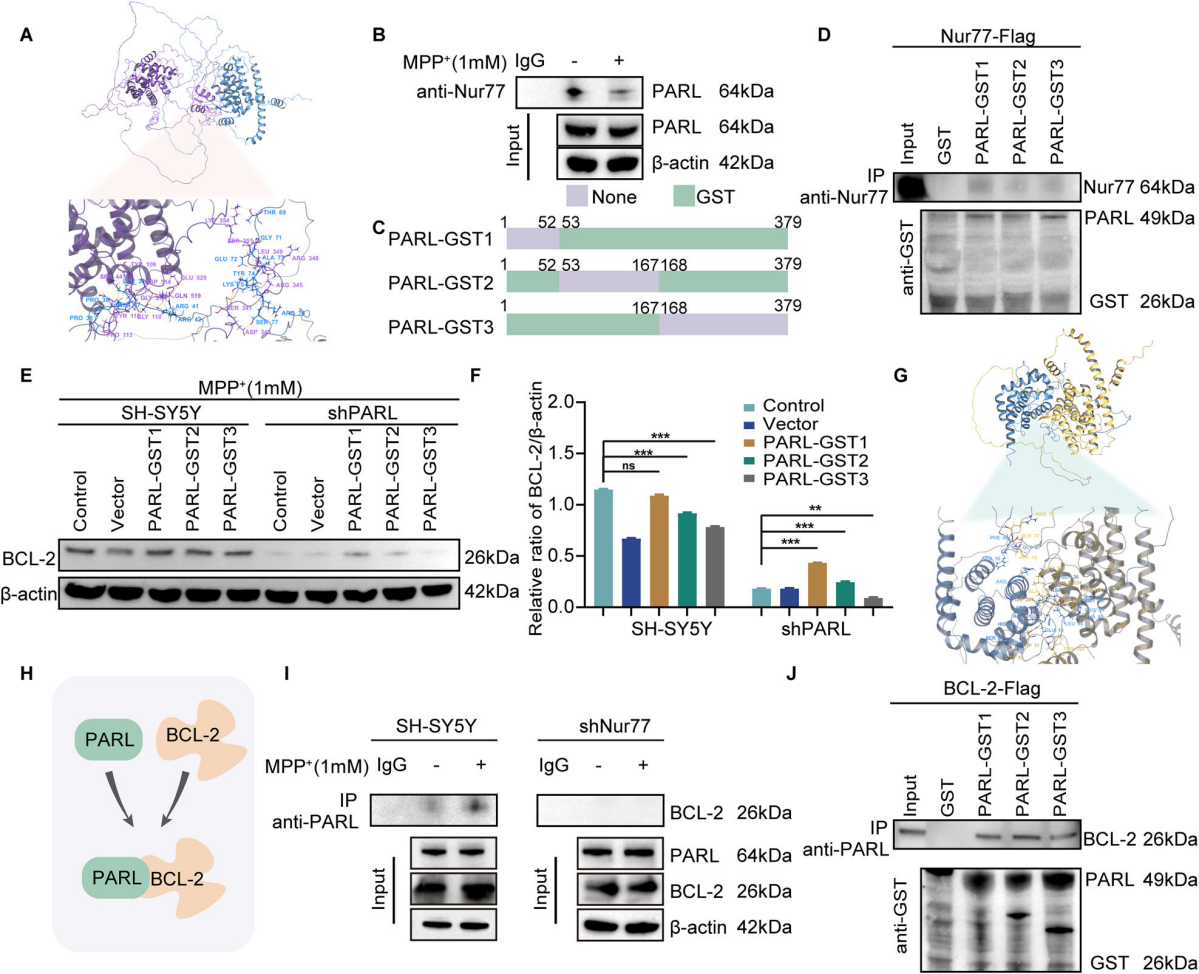
Nur77 promotes PARL-BCL-2 interaction to attenuate apoptosis in MPP^+^-treated SH-SY5Y cells. **A** Predicted Nur77-PARL binding interface by AlphaFold2. Nur77 (purple) interacts with PARL (blue) via key residues: salt bridge (Nur77-GLU525/PARL-ARG41), hydrogen bonds (Nur77-GLN519/PARL-ARG42; Nur77-ARG318/PARL-ARG9). **B** Co-immunoprecipitation (co-IP) validation of Nur77-PARL interaction in MPP^+^ (1 mM, 24 h)-treated SH-SY5Y cells. Lysates immunoprecipitated with anti-Nur77 antibody. IgG: isotype control. **C** Schematic of PARL truncations (GST-tagged fragments) used for binding assays. Numbers denote amino acid positions. **D** GST pull-down assay showing Nur77 binds preferentially to PARL-GST1 (1-167 aa). Input: 100% lysate; GST control: empty vector. **E**, **F** Western blot analysis of BCL-2 levels after PARL peptide inhibition. β-actin: loading control. **G** AlphaFold2-predicted PARL-BCL-2 complex. PARL (yellow) binds BCL-2 (blue) via hydrogen bonds (PARL-TRP158/BCL-2-GLU13; PARL-ARG76/BCL-2-SER50) and a π-π interaction (PARL-TRP11/BCL-2-HIS20). **H** Co-IP confirms PARL-BCL-2 interaction in MPP^+^-treated cells. Lysates immunoprecipitated with anti-PARL antibody. **I**, **J** GST pull-down assays demonstrate Nur77 knockdown disrupts PARL-BCL-2 binding. PARL-GST2 (1-167 aa) shows strongest BCL-2 affinity.

To delineate the molecular interplay between Nur77 and PARL in Parkinson’s disease (PD), we used AlphaFold2 to predict their three-dimensional structures. We then analyzed the predicted binding interfaces. Computational modeling revealed a stable interaction between Nur77 (purple) and PARL (blue), Specifically, GLU525 of Nur77 formed a salt bridge with ARG41 of PARL, while GLN519 of Nur77 formed two hydrogen bonds with ARG42 of PARL. Additionally, ARG318 of Nur77 formed a hydrogen bond with ARG9 of PARL (Fig. 7A). Co-immunoprecipitation assays in MPP^+^-treated SH-SY5Y cells validated this interaction (Fig. 7B), supporting AlphaFold2’s accuracy predictions for protein-protein interface.

To pinpoint critical interaction domains, we designed GST-tagged PARL fragments (Fig. 7C). GST pull-down assays demonstrated that Nur77 mainly bound PARL-GST1,a region overlapping with binding motifs predicted by AlphaFold2-predicted (Fig. 7D). Functional studies showed that inhibiting PARL-GST1 reduced BCL-2 expression. This finding suggests that PARL plays a role in stabilizing this anti-apoptotic protein (Fig. 7E, F).

To further explore the PARL-BCL-2 interaction, we modeled their binding interface using AlphaFold2. The predicted structure revealed a stable complex between PARL (yellow) and BCL-2 (blue). Key interactions include a hydrogen bond between TRP158 of PARL and GLU13 of BCL-2, hydrogen bonds between ARG76 of PARL and SER50 of BCL-2, and a π-π interaction between TRP11 of PARL and HIS20 of BCL-2 (Fig. 7G). Co-IP and GST pull-down confirmed these interactions (Fig. 7H), with PARL-GST2 (1-167 aa) exhibiting 2.3-fold stronger BCL-2 binding than other fragments (Fig. 7I, J). Silencing Nur77 disrupted PARL-BCL-2 binding (Fig. 7I, J), demonstrating Nur77’s role as a scaffold for this complex. These findings demonstrate AlphaFold’s utility in identifying critical residues for functional interactions. They also highlight Nur77-PARL-BCL-2 as a novel anti-apoptotic axis in PD

## Discussion

Our study establishes PARL as a central regulator of mitochondrial apoptosis in PD and bridges clinical observations with mechanistic insights into dopaminergic neuron survival. The discovery of reduced serum PARL levels in PD patients, correlating with motor and cognitive decline, identifies PARL as both a novel diagnostic biomarker and a active driver to disease progression. This finding shifts PD biomarker research from focusing on α-synuclein or inflammatory markers to mitochondrial quality control proteins, enabling earlier and more precise diagnosis.

PARL is nvolved in several cellular processes, including mitophagy, ferroptosis, and other signal transduction and cellular quality control mechanisms within its transmembrane domain [39–41]. Previous research has highlighted that PARL downregulation diminishes HtrA2 processing, thereby increasing the vulnerability of neurons [42]. A genome-wide survival analysis identified PARL as a novel locus associated with clinical progression and neurodegeneration in Alzheimer’s disease [43].

This finding highlights the role of PARL in neurodegenerative diseases. However the specific implications of PARL particularly its interactions with the BCL-2 family in apoptotic pathways. remain underexplored and represent an unknown area in PD. Our work uncovers a non-enzymatic mechanism whereby PARL stabilizes the balance between BCL-2 and BAX homeostasis via direct interaction with BCL-2. Several studies have explored the relationship between PARL and apoptosis. For example, PARL regulates the release of cytochrome c during apoptosis through remodeling the OPA1 crest, and mature Smac alone can induce apoptosis in PARL ^−/−^ cells [44]. PARL interacts with BCL-2 family-related protein HAX1, inhibiting apoptosis in lymphocytes through proteolytic activating Omi/HtrA2 proteolytically and suppressing active BAX [16, 45].

Other studies have primarily focused on the fact that PARL deficiency leads to early mitochondrial ultrastructural abnormalities in neurons, followed by neuronal necrosis, a finding consistent with our observations. However, these studies suggest that neuronal death is not directly associated with apoptosis. Nonetheless, it is undeniable that apoptosis occurs in neurodegenerative diseases as well as in immune organ diseases, such as those affecting the thymus and spleen [46]. Therefore, the exact role of PARL in neurodegenerative diseases remains unclear and requires further study. Our study demonstrates that PARL can regulate apoptosis in PD through BCL-2, thereby exerting a protective effect [18, 20].

Our results showed that the *PARL*-knockdown decreases BCL-2 levels through high-throughput mRNA sequencing analysis. In vitro and in vivo studies also demonstrated that PARL alleviated the apoptosis of dopaminergic neurons in PD through a BCL-2 mediated anti-apoptotic pathway. This indicates that PARL inhibits mitochondrial apoptosis by maintaining BCL-2/BAX homeostasis. Moreove, further investigations demonstrated that PARL’s neuroprotective effects depended on its physical interaction with BCL-2. Although PARL is classically known to regulate mitochondrial dynamics through substrate cleavage (e.g., PGAM5 or PHB2), our study identifies a non-enzymatic, protein interaction-dependent mechanism by which PARL stabilizes BCL-2. This finding expands the functional role of PARL, highlighting its multifaceted role in mitochondrial homeostasis.

We have previously shown that Nur77 translocation from cytoplasm to mitochondria and enhances mitochondrial autophagy mediated by the mitochondrial endosomal gene PHB2 in PD [37]. In this study, we revealed that Nur77 translocated from cytoplasm to mitochondria, interacts with PARL, enhanceed interaction between PARL and BCL-2, increased BCL-2 expression and decreased BAX and cleaved Caspase3 expression, and inhibits dopaminergic neuron apoptosis. Studies in the PD model showed that *Nur77* knockdown decreased the protein expression of PARL and BCL-2, Conversely, overexpression of Nur77 increased the expression of PARL and BCL-2 proteins levels, which uncover that Nur77 enhances the interaction between PARL and BCL-2 to play an anti-apoptotic role in MPP^+^-injured SH-SY5Y cells. This process likely involves direct binding of Nur77 to the PARL promoter, however, precise regulatory elements remain to be validated. Our data further reveal that the neuroprotective effects of the orphan nuclear receptor Nur77 are mediated, at least partially, through PARL.

Our data reconcile these dual roles by demonstrating that PARL’s anti-apoptotic function dominates in PD models, In these models, its interaction with BCL-2 prevents mitochondrial outer membrane permeabilization (MOMP) and caspase-3 activation. Nur77 acts as a molecular bridge, strengthening PARL-BCL-2 interactions while transcriptionally upregulating PARL expression. The Nur77 agonist Csn-B rescues BCL-2/BAX balance in wild-type models, underscoring PARL’s indispensability in this pathway. This axis represents a paradigm shift in understanding Nur77’s anti-apoptotic effects, which were previously attributed solely to BCL-2 transcriptional regulation. Instead, we reveal a post-translational mechanism whereby Nur77 stabilizes BCL-2 via PARL, bypassing direct interactions with yhe promoter.

The discovery of the Nur77-PARL-BCL-2 pathway therefore opens new avenues for PD therapeutics. Enhancing PARL activity via Nur77 agonists or *PARL*-targeted gene therapy could restore mitochondrial homeostasis and inhibit apoptosis. Enhancing PARL activity—via small-molecule Nur77 activators (such as cytosporone B) or *PARL*-targeted gene therapy—could restore mitochondrial homeostasis. Our structural mapping of PARL’s and BCL-2-binding domain (1-167 aa) identifies druggable pockets for small-molecule design, akin to venetoclax’s targeting of BCL-2 in cancer. Additionally, serum PARL levels may serve as a dynamic biomarker for PD progression and treatment response, complementing current α-synuclein-based assays. Longitudinal studies are warranted to validate serum PARL as a biomarker for PD progression and treatment response in diverse PD cohorts.

### Limitations and future directions

While MPTP/MPP^+^ models replicate acute mitochondrial dysfunction, they incompletely mimic chronic neurodegeneration in human PD; therefore, validation in patient-derived neurons (e.g., iPSC-midbrain organoids) is critical. The precise Nur77-binding elements in the PARL promoter need identification through CRISPR-Cas9 screening or ChIP-seq validation.

## Conclusion

By integrating structural biology (AlphaFold2), multi-omics, and functional assays, we redefine PARL as a linchpin of mitochondrial apoptosis regulation in PD. The Nur77-PARL-BCL-2 axis offers a therapeutically target pathway to delay dopaminergic neurodegeneration. These findings not only deepen our understanding of apoptosis in PD but also provide a theoretical foundation for the development of neuroprotective therapies targeting the Nur77-PARL axis. Future research should prioritize screening small-molecule modulators of this pathway and evaluating their efficacy across different PD stages.

## Supplementary information

Supplementary figures

Supplementary figure legends

Unedited blot and gel images

## Data Availability

The datasets generated and/or analysed during the current study are available from the corresponding authors on reasonable request.
