# Supplementary figures and images for: PARL stabilizes mitochondrial BCL-2 via Nur77-mediated scaffolding as a therapeutic strategy for Parkinson’s disease
Source: Cell Death Dis. 2025 Oct 6;16(1):700. doi: 10.1038/s41419-025-08035-8 (PMC12501375; doi:10.1038/s41419-025-08035-8)

A

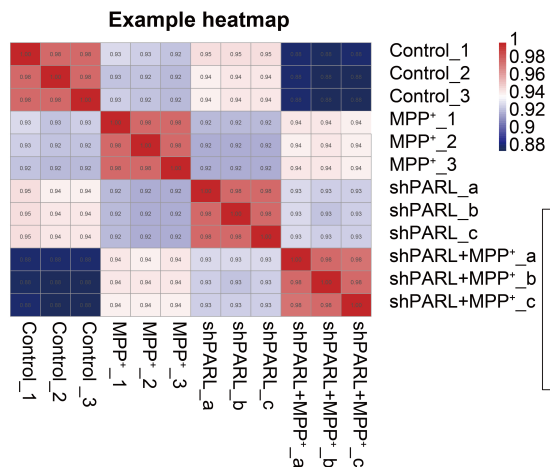

B

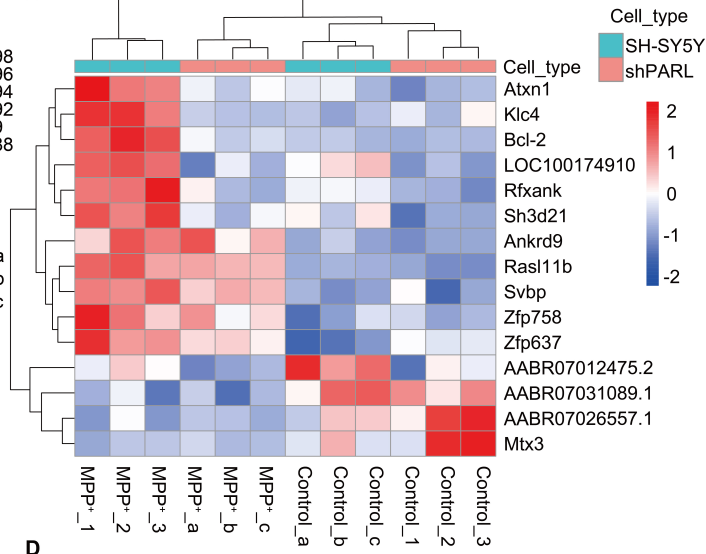

C

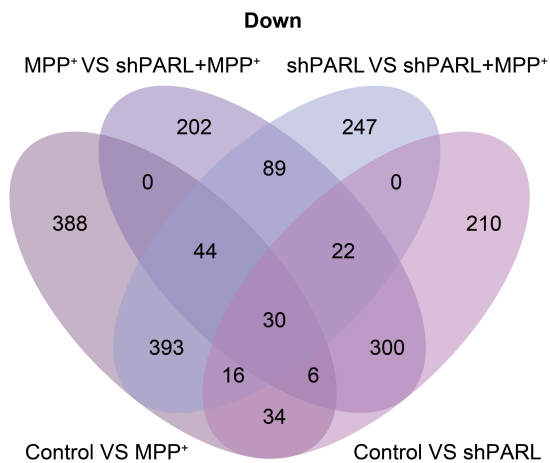

D

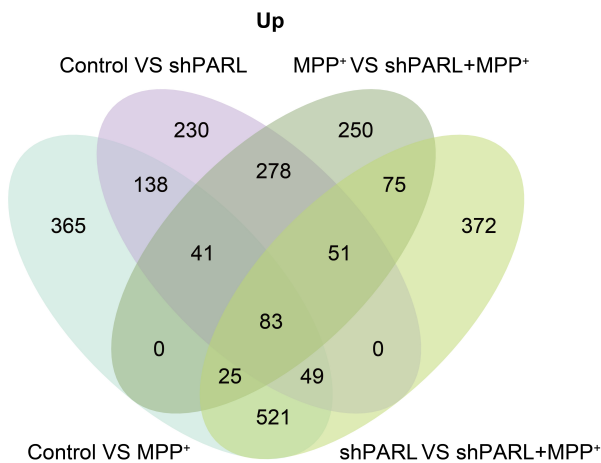

E

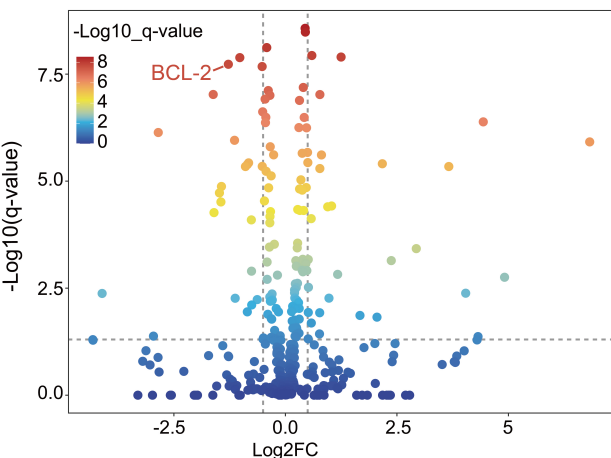

F

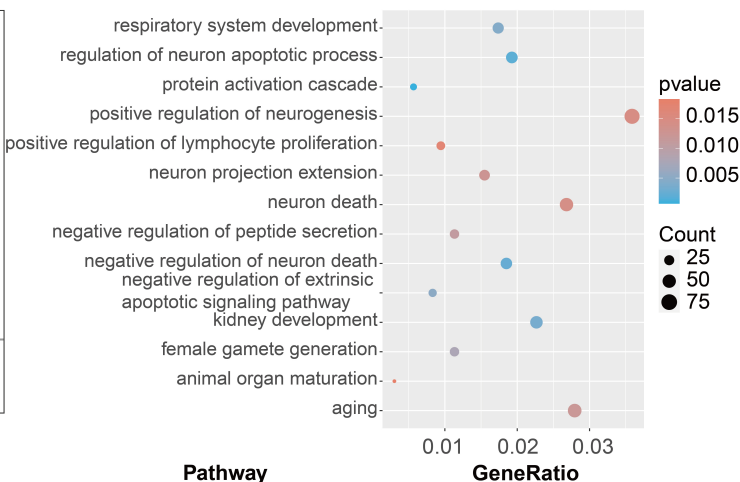

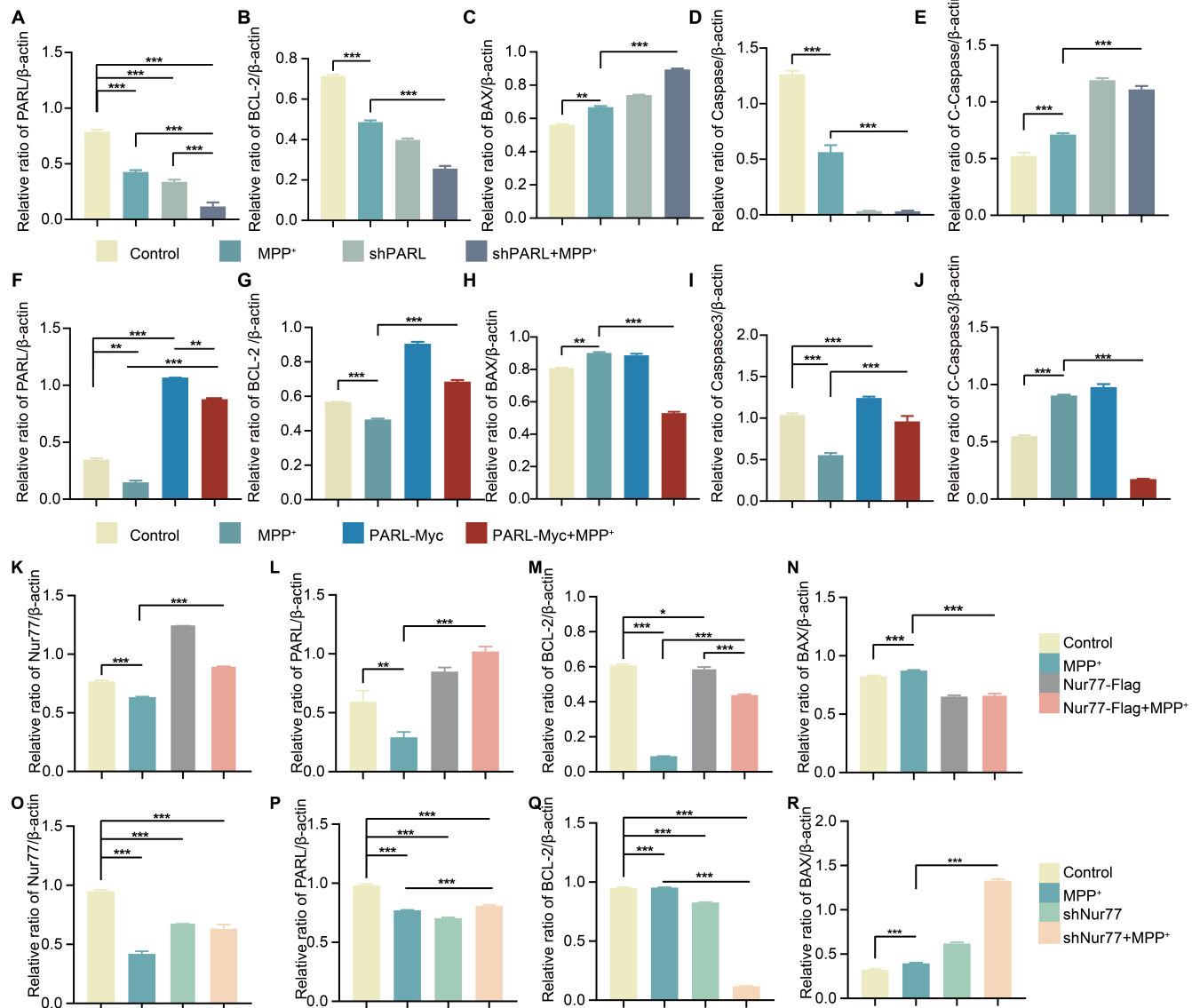

**A**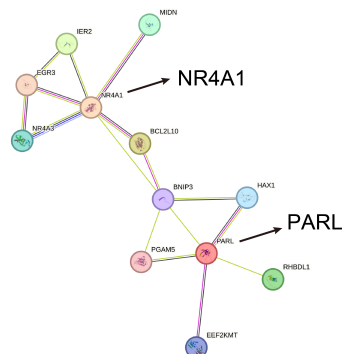**B**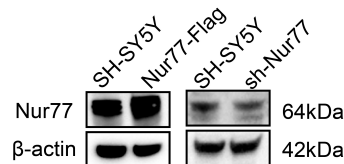**C**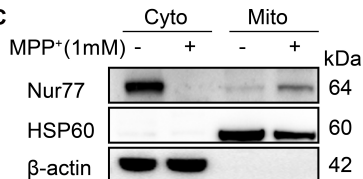**D**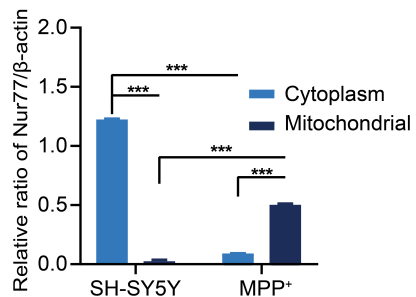**E**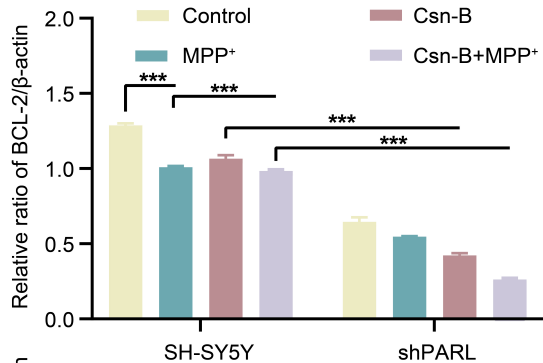**F**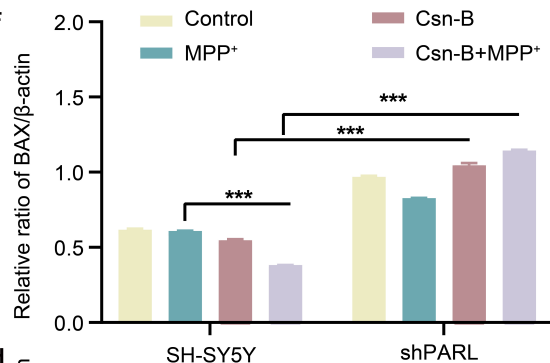**G**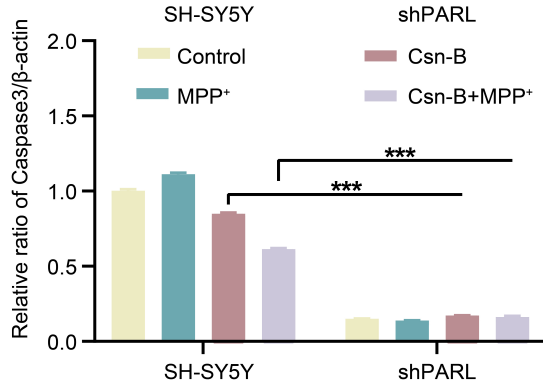**H**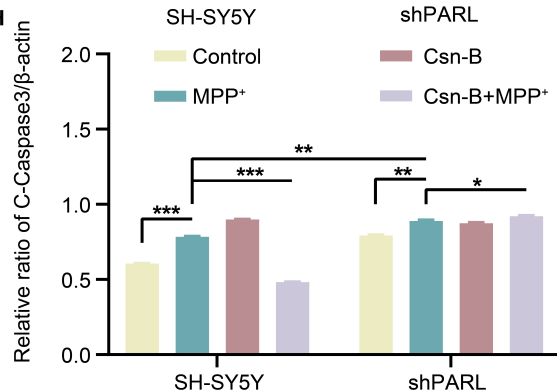

**A**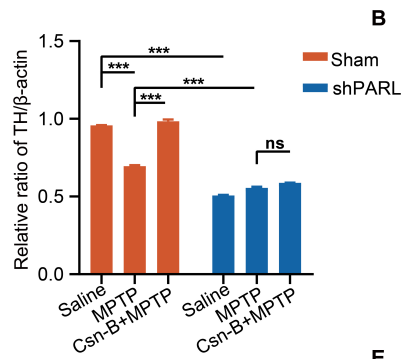**B**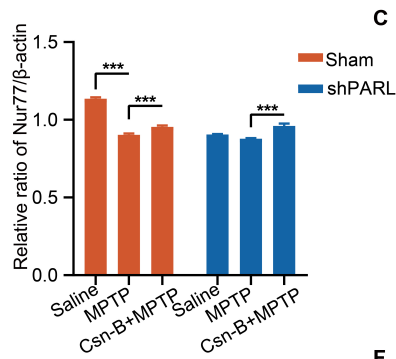**C**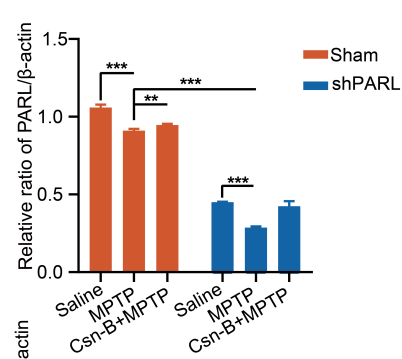**D**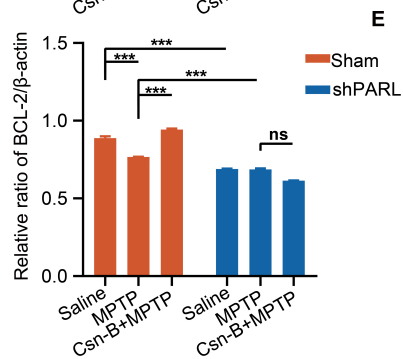**E**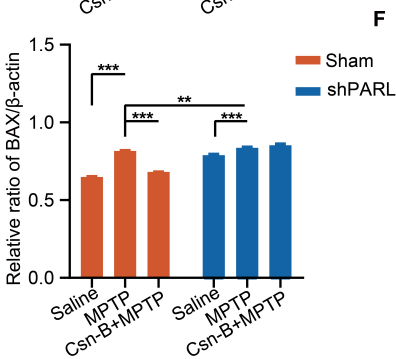**F**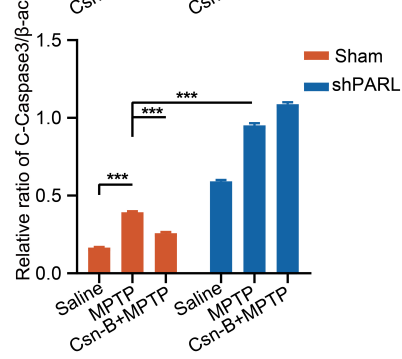

Supplement: Supplementary file 1 — Supplementary figures [file 41419_2025_8035_MOESM1_ESM.pdf]

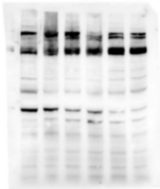

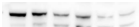

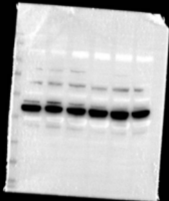

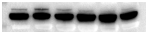

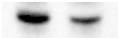

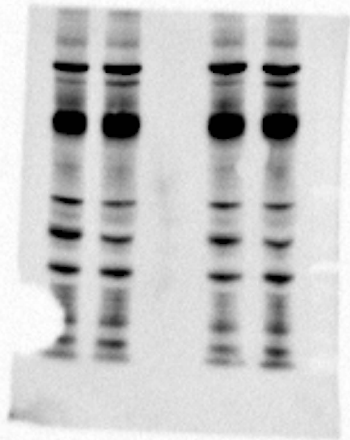

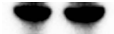

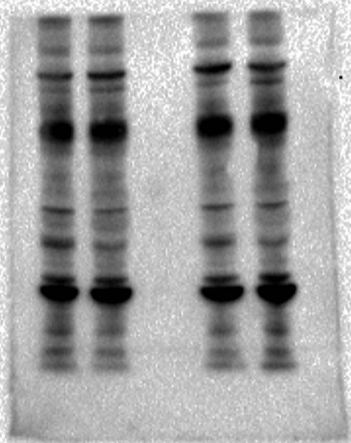

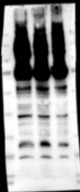

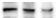

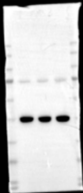

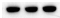

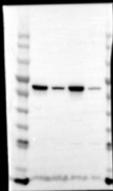

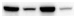

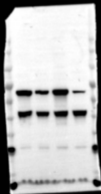

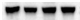

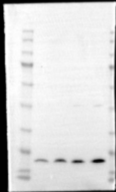

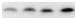

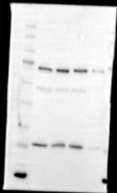

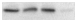

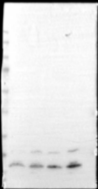

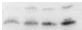

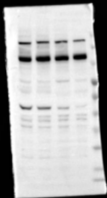

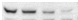

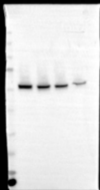

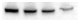

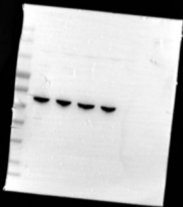

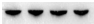

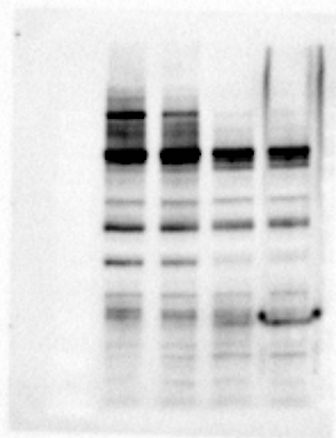

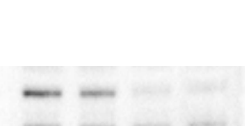

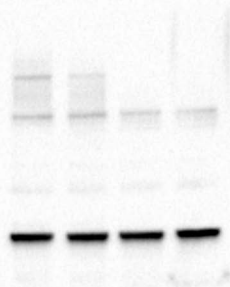

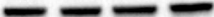

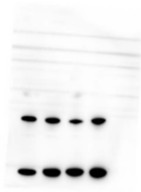

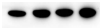

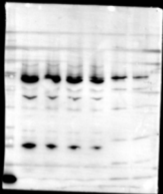

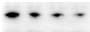

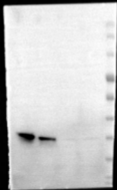

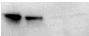

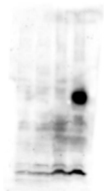

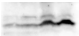

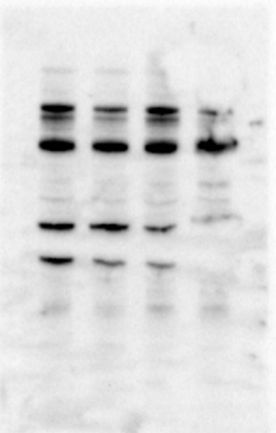

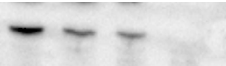

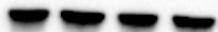

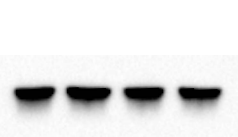

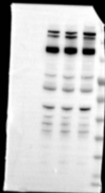

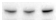

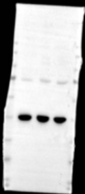

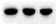

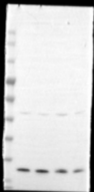

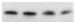

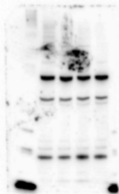

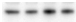

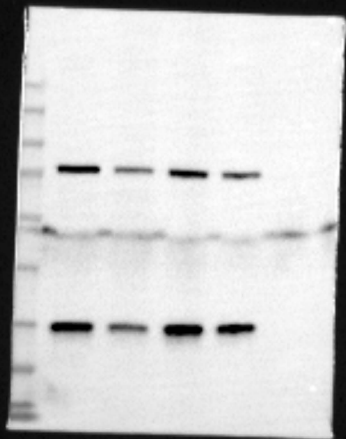

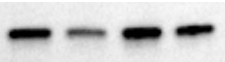

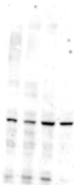

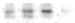

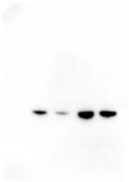

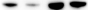

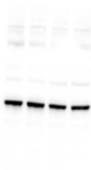

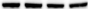

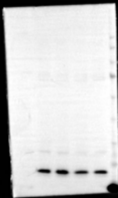

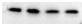

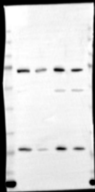

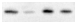

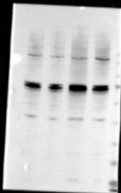

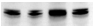

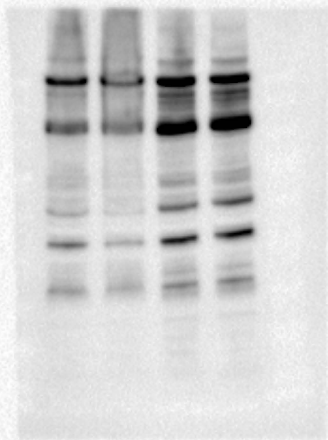

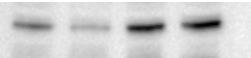

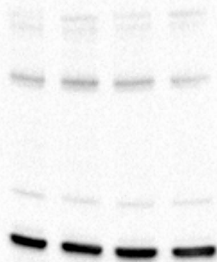

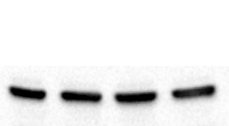

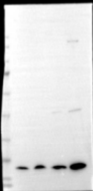

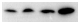

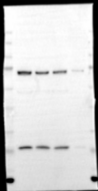

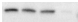

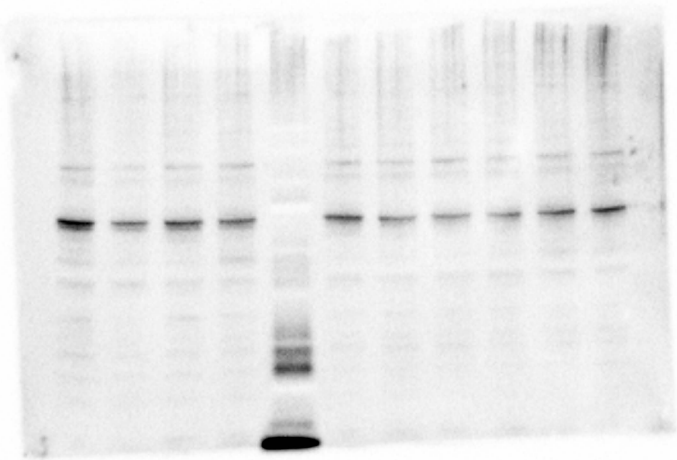

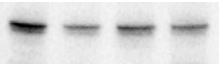

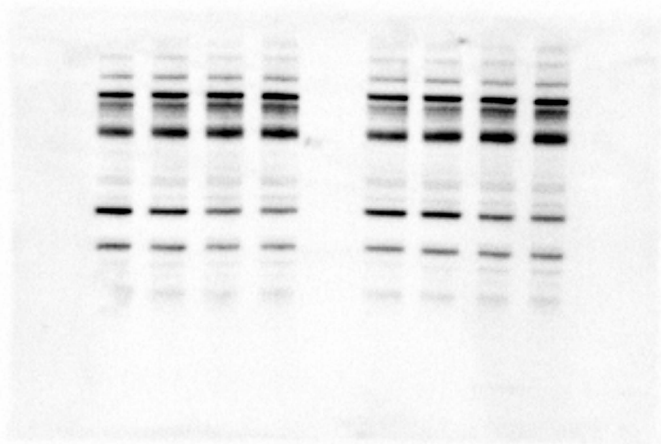

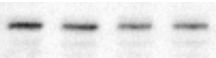

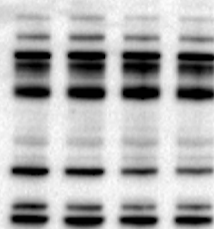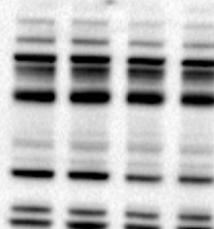

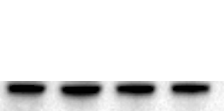

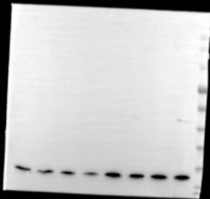

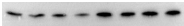

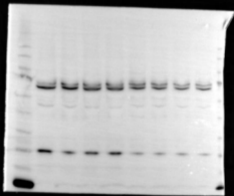

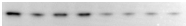

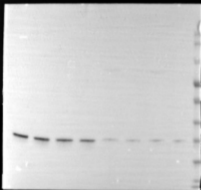

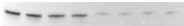

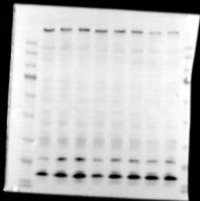

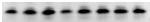

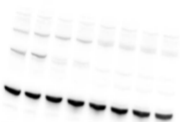

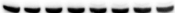

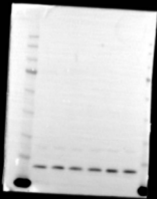

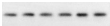

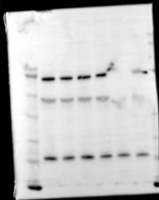

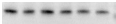

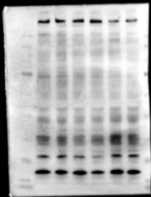

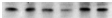

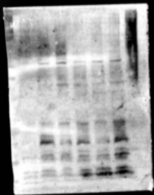

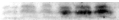

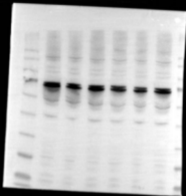

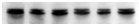

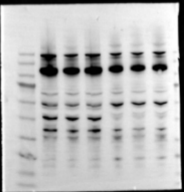

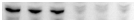

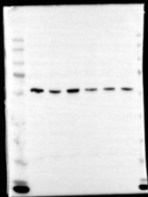

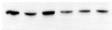

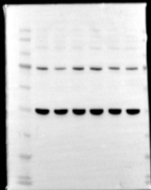

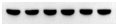

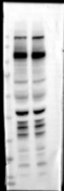

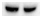

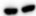

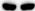

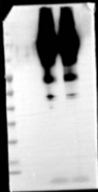

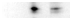

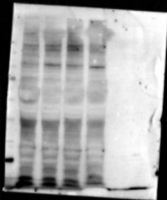

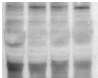

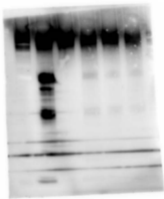

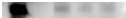

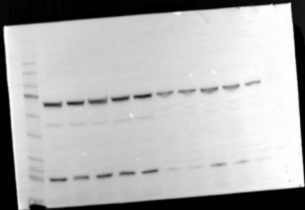

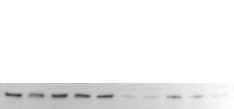

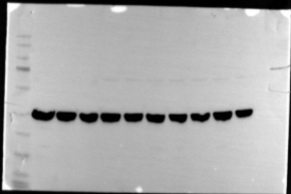

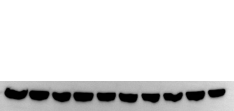

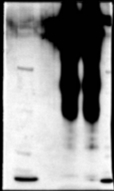

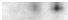

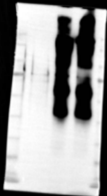



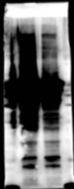

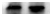

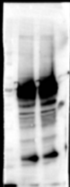

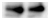

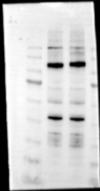

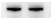

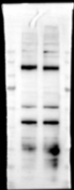

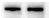

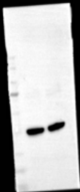

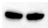

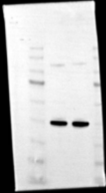

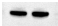

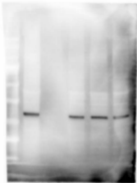

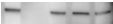

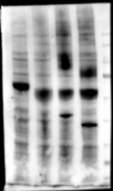

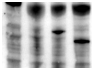

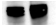

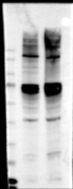

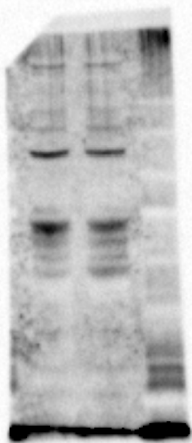

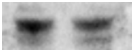

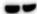

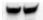

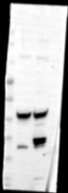

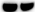

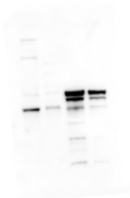

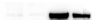

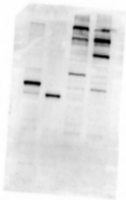

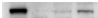

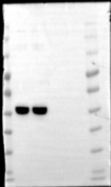

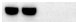

Supplement: Supplementary file 3 — Unedited blot and gel images [file 41419_2025_8035_MOESM3_ESM.pdf]
