# Supplementary material for: PARL stabilizes mitochondrial BCL-2 via Nur77-mediated scaffolding as a therapeutic strategy for Parkinson’s disease
Source: Cell Death Dis. 2025 Oct 6;16(1):700. doi: 10.1038/s41419-025-08035-8 (PMC12501375; doi:10.1038/s41419-025-08035-8)
Supplement: Supplementary file 2 — Supplementary figure legends [file 41419_2025_8035_MOESM2_ESM.docx]

**Supplementary Figure 1.** **High-Throughput mRNA Sequencing Reveals PARL Knockdown Exacerbates Apoptosis in MPP⁺-Treated SH-SY5Y Cells.**

(A) Pearson correlation matrix demonstrates high inter-replicate consistency for shPARL groups. Reduced correlation in the shPARL+MPP⁺ group suggests gene expression dysregulation.

(B) Hierarchical clustering heatmap (Z-score normalized) reveals 328 genes with >2-fold changes in shPARL+ MPP⁺ vs. MPP⁺-alone groups, including significant upregulation of apoptosis-related genes.

(C and D) Venn diagram identifies 512 core differentially expressed genes (DEGs) responsive to both PARL knockdown and MPP⁺ neurotoxicity (intersection). Apoptosis-related genes account for 37% of enriched terms.

(E) Volcano plot confirms mRNA-protein consistency: 83% of apoptosis pathway-related DEGs show significant changes at the protein level (Benjamini-Hochberg adjusted *p* <0.01).

(F) GO enrichment analysis. Biological process enrichment analysis of differentially expressed proteins between MPP^+^ and shPARL+MPP^+^.

**Supplementary Figure 2.**  **Protein quantitative analysis in different treatment SH-SY5Y cells**

(A-E) Quantitative analysis of PARL, BCL-2, BAX, Caspase3, and C-Caspase3 protein expressions in SH-SY5Y and Knockdown PARL cells treated with MPP**^+^**.

(F-J) Quantitative analysis of PARL, BCL-2, BAX, Caspase3, and C-Caspase3 protein expressions in SH-SY5Y and PARL-Myc cells treated with MPP**^+^**.

(K-N) Quantitative analysis of Nur77, PARL, BCL-2 and BAX protein expressions in SH-SY5Y and Nur77-Flag cells treated with MPP**^+^**.

(O-R) Quantitative analysis of Nur77, PARL, BCL-2 and BAX protein expressions in SH-SY5Y and shNur77 cells treated with MPP**^+^**.

**Supplementary Figure 3.** **STRING prediction and Protein quantitative analysis**

(A) STRING prediction of protein-protein interactions.

(B) SH-SY5Y cells transfected with shRNA-Nur77 and Nur77-Flag. And Western blot was used to verify the expression of Nur77 in SH-SY5Y cells.

(C) Western blot showing Nur77 levels in mitochondrial extract from SH-SY5Y cells and MPP^+^ treated cells.

(D) Quantitative analysis of Nur77 expressions in cytoplasm and mitochondrial in SH-SY5Y cells treated with or without MPP^+^.

(E-H) Quantitative analysis of BCL-2, BAX, Caspase3 and Cleaved-Caspase3 protein expressions in different Groups.

(ns, not significant, ∗p< 0.05, ∗∗p< 0.01, ∗∗∗p< 0.001 compared with the control group; n =3, mean ± SEM).

**Supplementary Figure 4.** **Protein quantitative analysis in PD mice**

(A-F) Quantitative analysis of TH, Nur77, PARL, BCL-2, BAX and Cleaved-Caspase3 protein expressions in different Groups.
